# Supplementary figures and images for: Diversified regulation of circadian clock gene expression following whole genome duplication
Source: PLoS Genet. 2020 Oct 8;16(10):e1009097. doi: 10.1371/journal.pgen.1009097 (PMC7575087; doi:10.1371/journal.pgen.1009097)

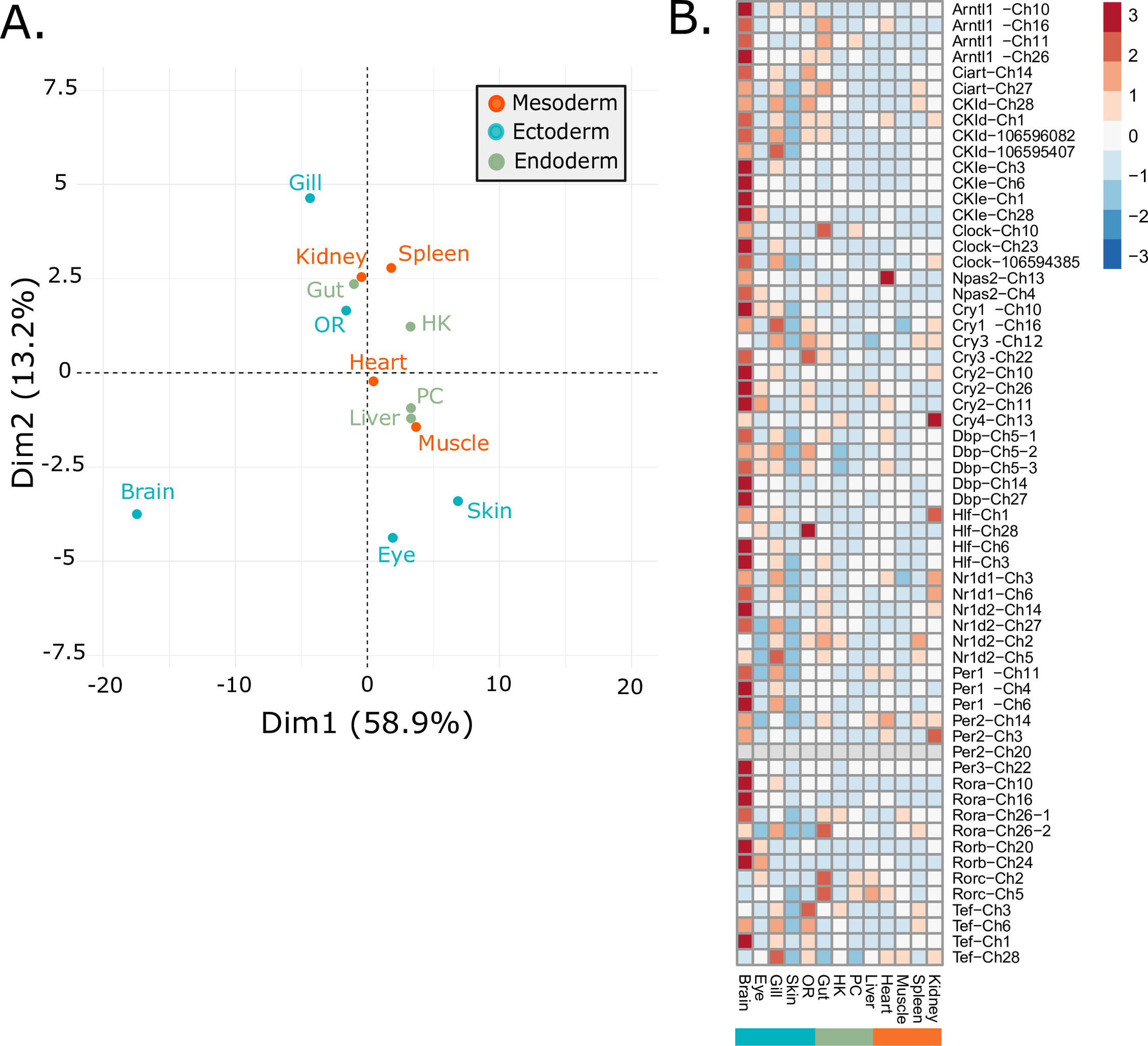

Supplement: S1 Fig — A. PCA plot showing the relative tissue differences when considering clock ohnologue expression. B. Heatmap showing the tissue specific expression of clock ohnologues. (TIF) [file pgen.1009097.s001.tif]

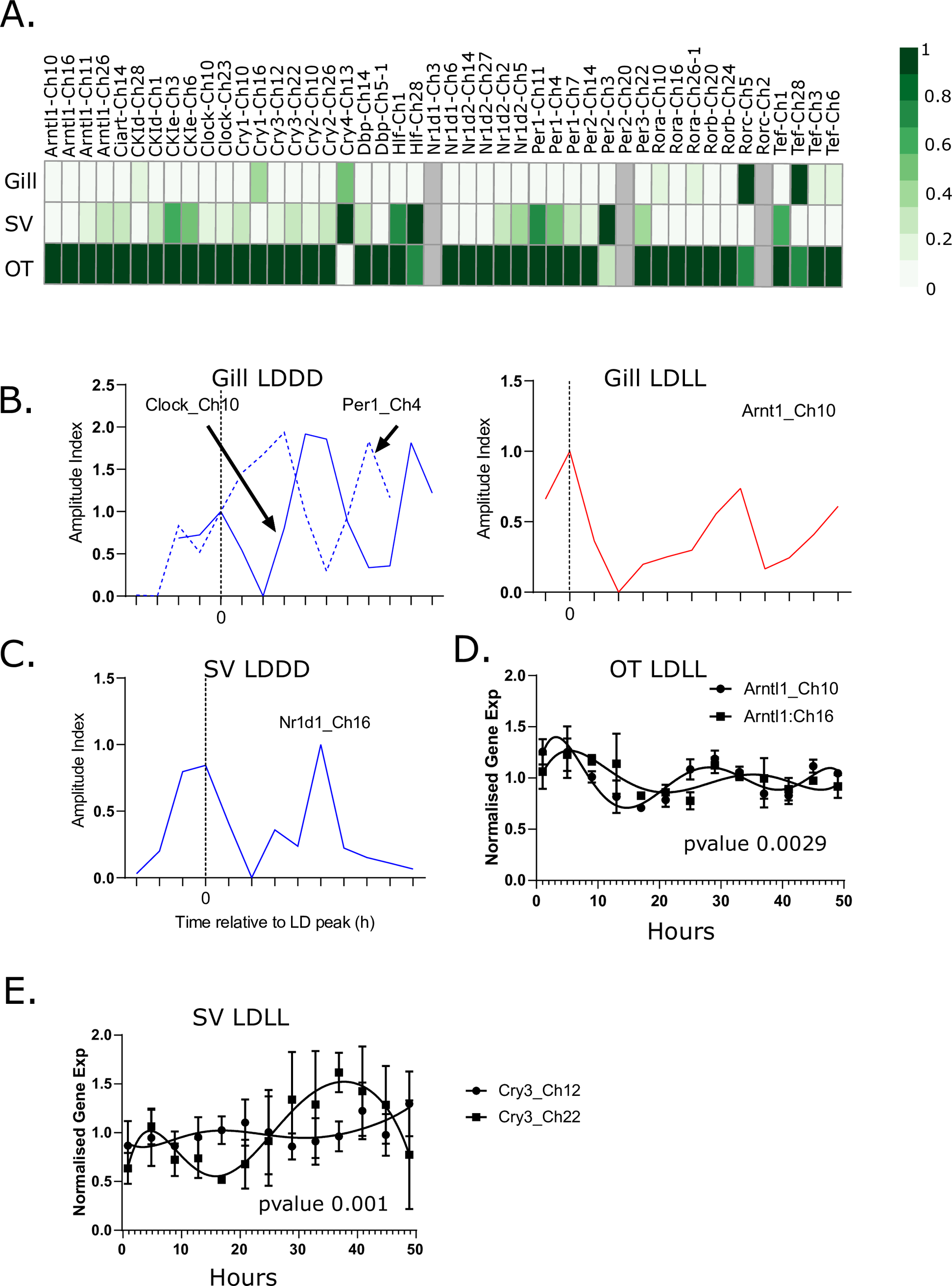

Supplement: S2 Fig — A. Heatmap showing the mesor expression for each clock ohnologue in three tissues. Grey indicates the gene is not expressed. B. Phase aligned plots for the gill. C. Phase aligned plot for the SV. D. Arntl1-Ch10/16 comparison: plot of non-linear regression using a sixth-order centered polynomial to fit the data and compare individual curves. P-value is the result of extra sum-of-squares F test. E. As above for Cry3-Ch12/22. (TIF) [file pgen.1009097.s002.tif]

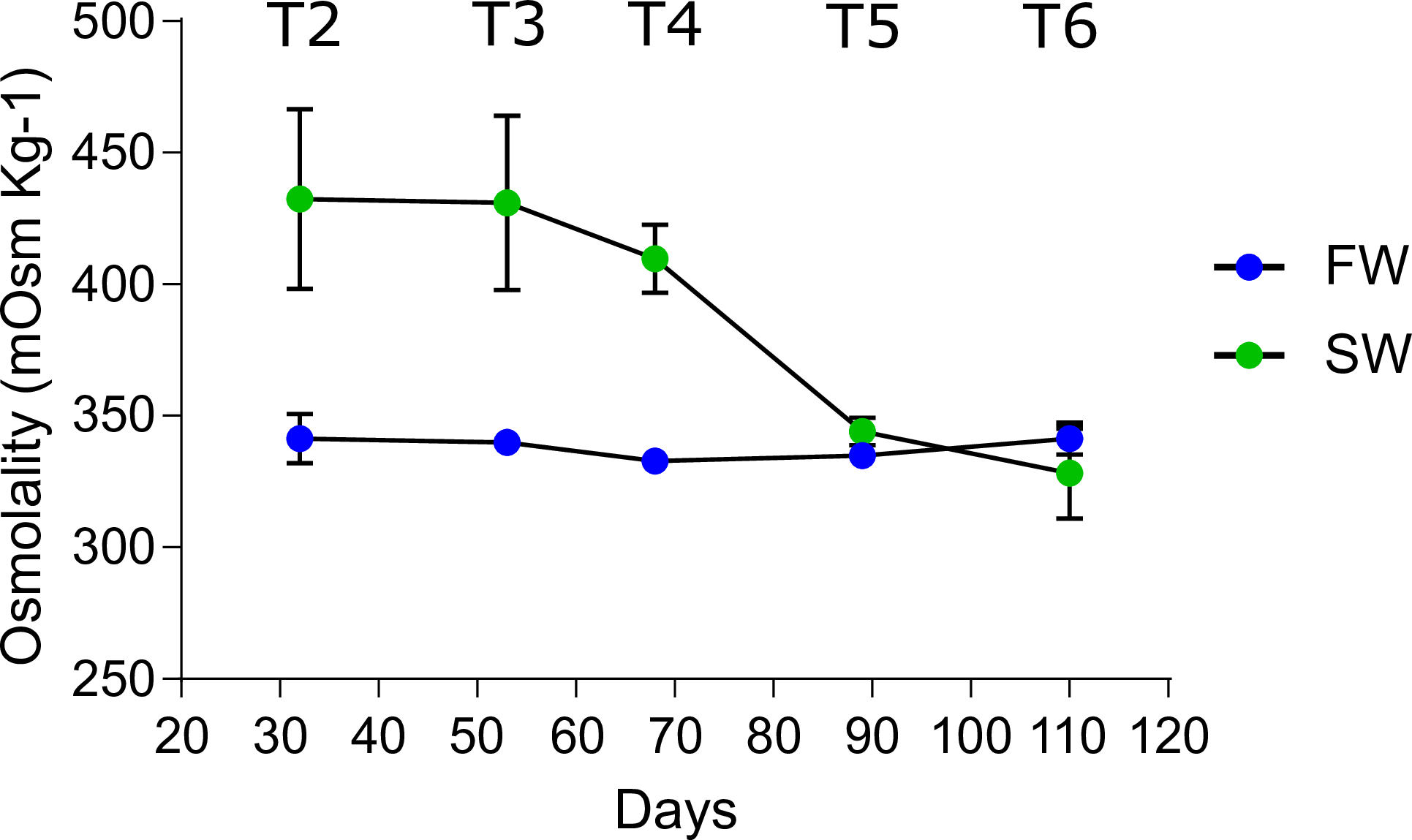

Supplement: S3 Fig — Osmolality (mOsm kg-1) is displayed for fish in freshwater (FW—blue) and seawater (SW—green) (n = 6). This plot show osmoregulatory capacity develops by the two latest timepoints (T5 and T6). (TIF) [file pgen.1009097.s003.tif]

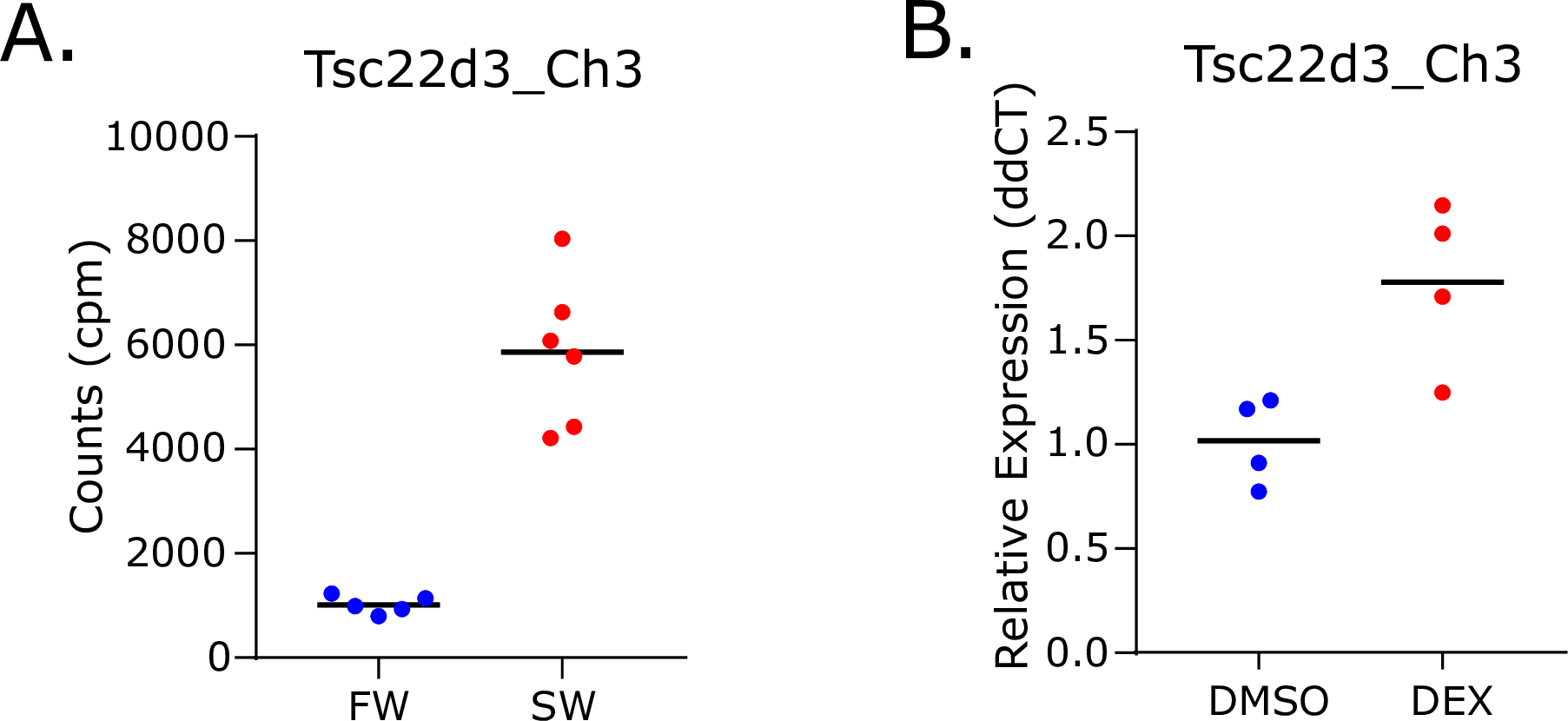

Supplement: S4 Fig — A. Gene expression of Tsc22dd3-Ch3 in vivo sea-water stress experiment (RNAseq counts per million (cpm)) and B. in vitro dexamethasone treatment (qPCR). (TIF) [file pgen.1009097.s004.tif]

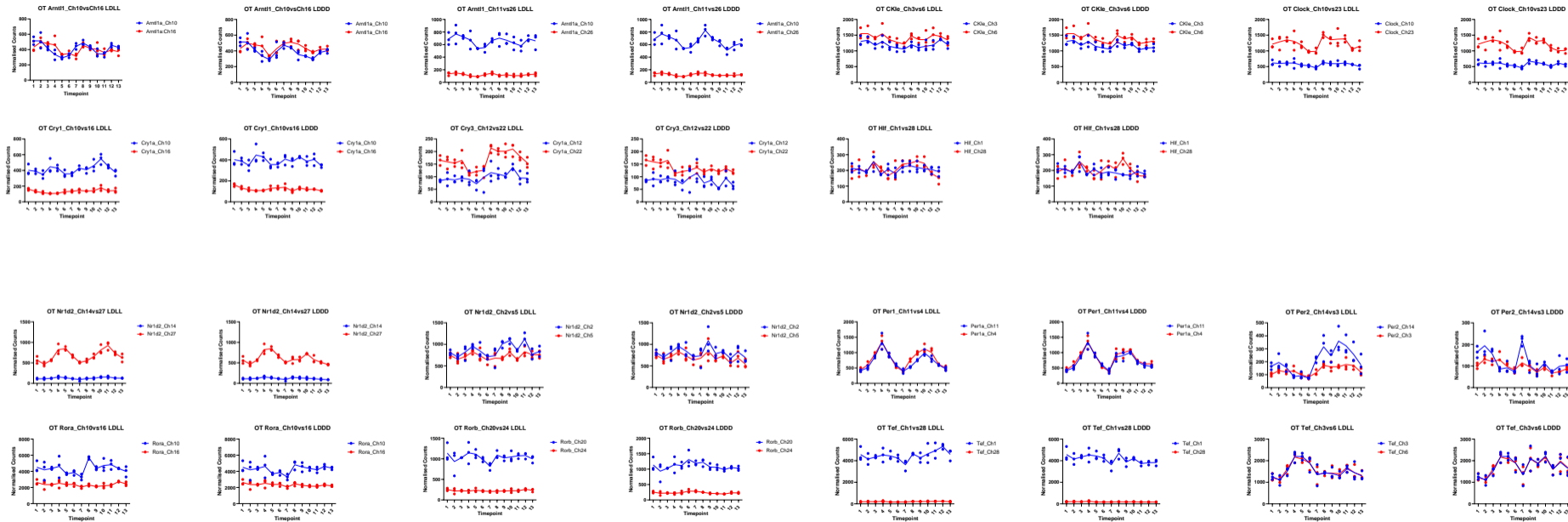

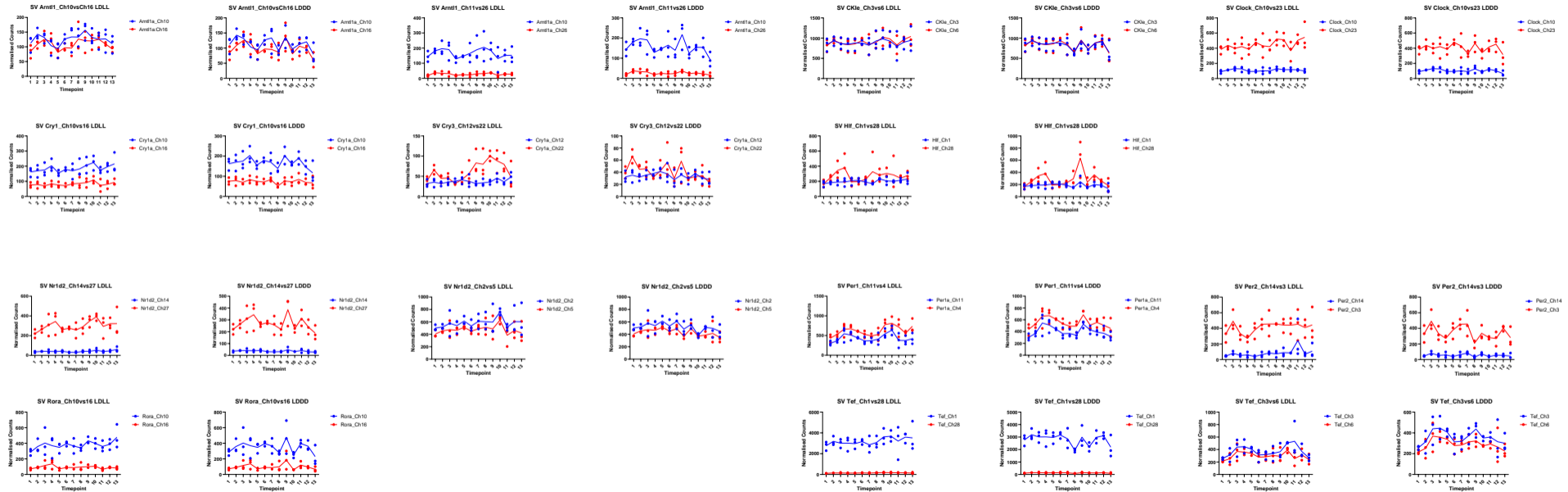

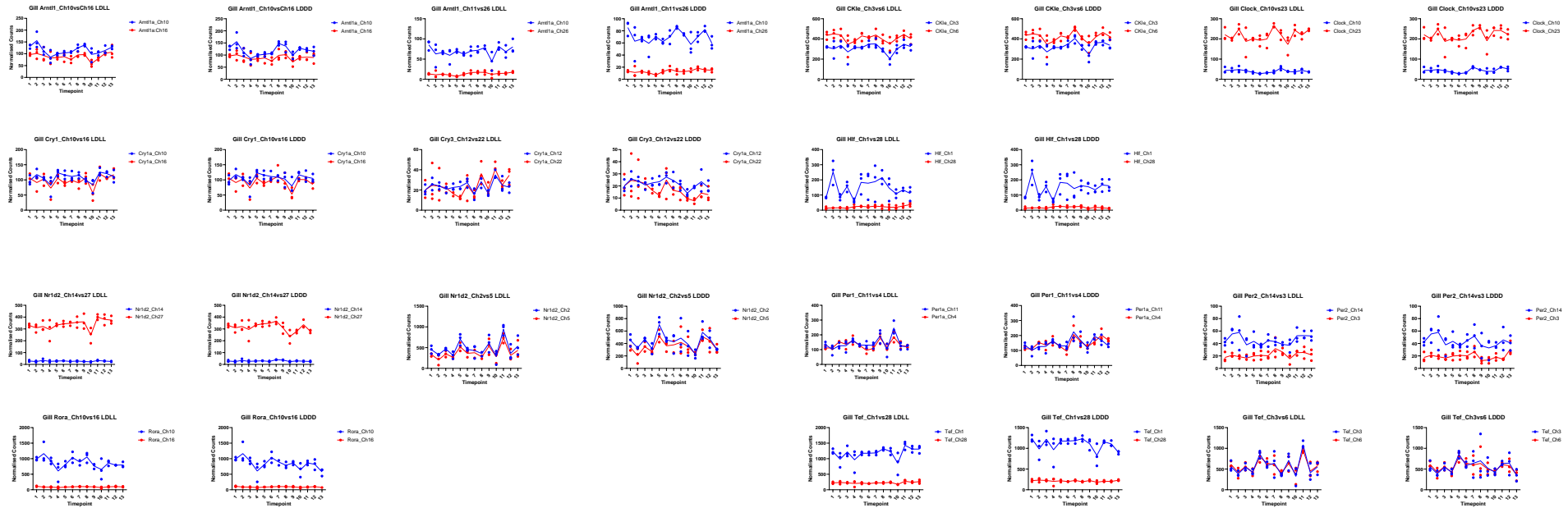

Supplement: S2 Appendix — (PDF) [file pgen.1009097.s012.pdf]
