# Supplementary material for: Diversified regulation of circadian clock gene expression following whole genome duplication
Source: PLoS Genet. 2020 Oct 8;16(10):e1009097. doi: 10.1371/journal.pgen.1009097 (PMC7575087; doi:10.1371/journal.pgen.1009097)

OG1v0000277 CKId/e

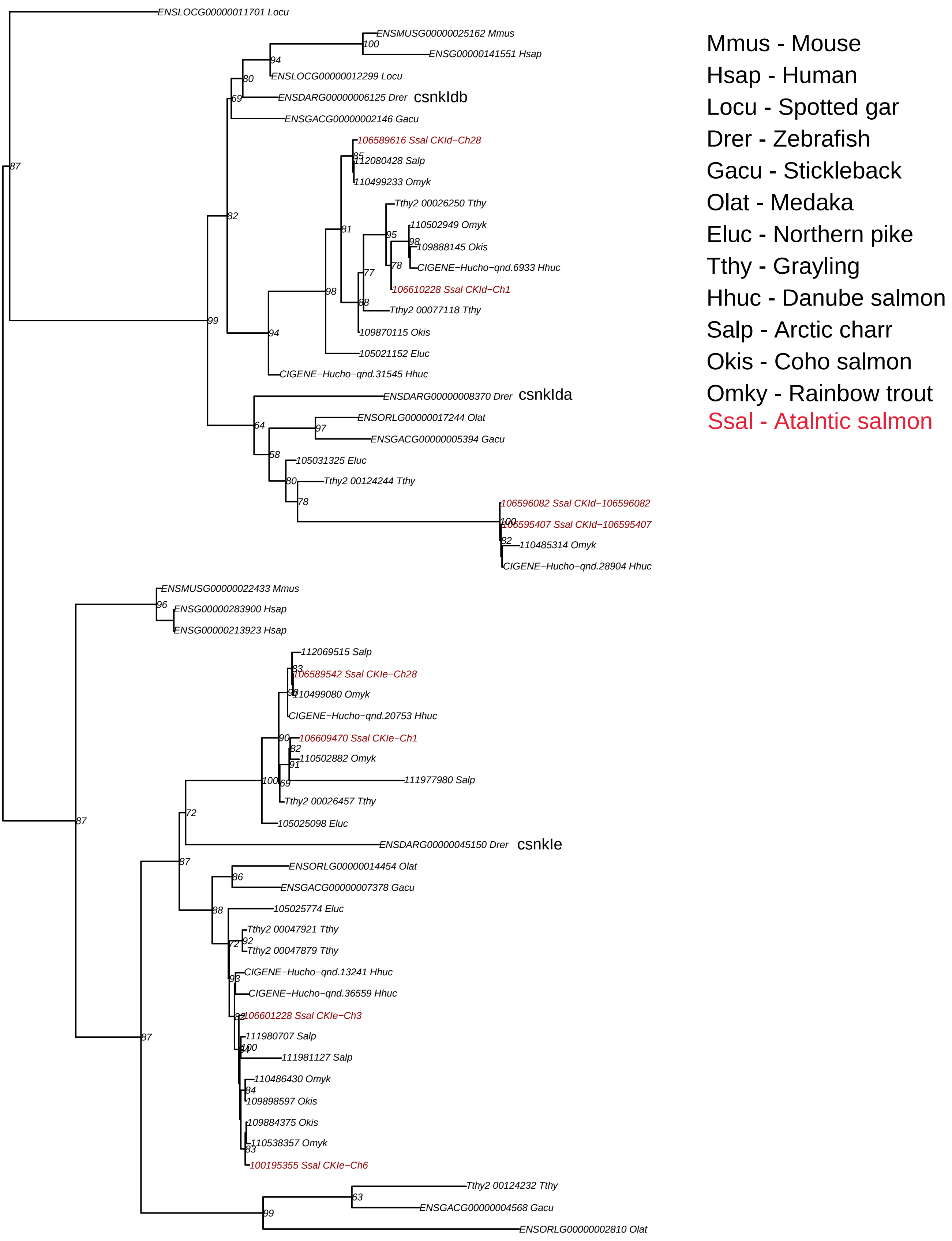

OG1v0000304 CRY

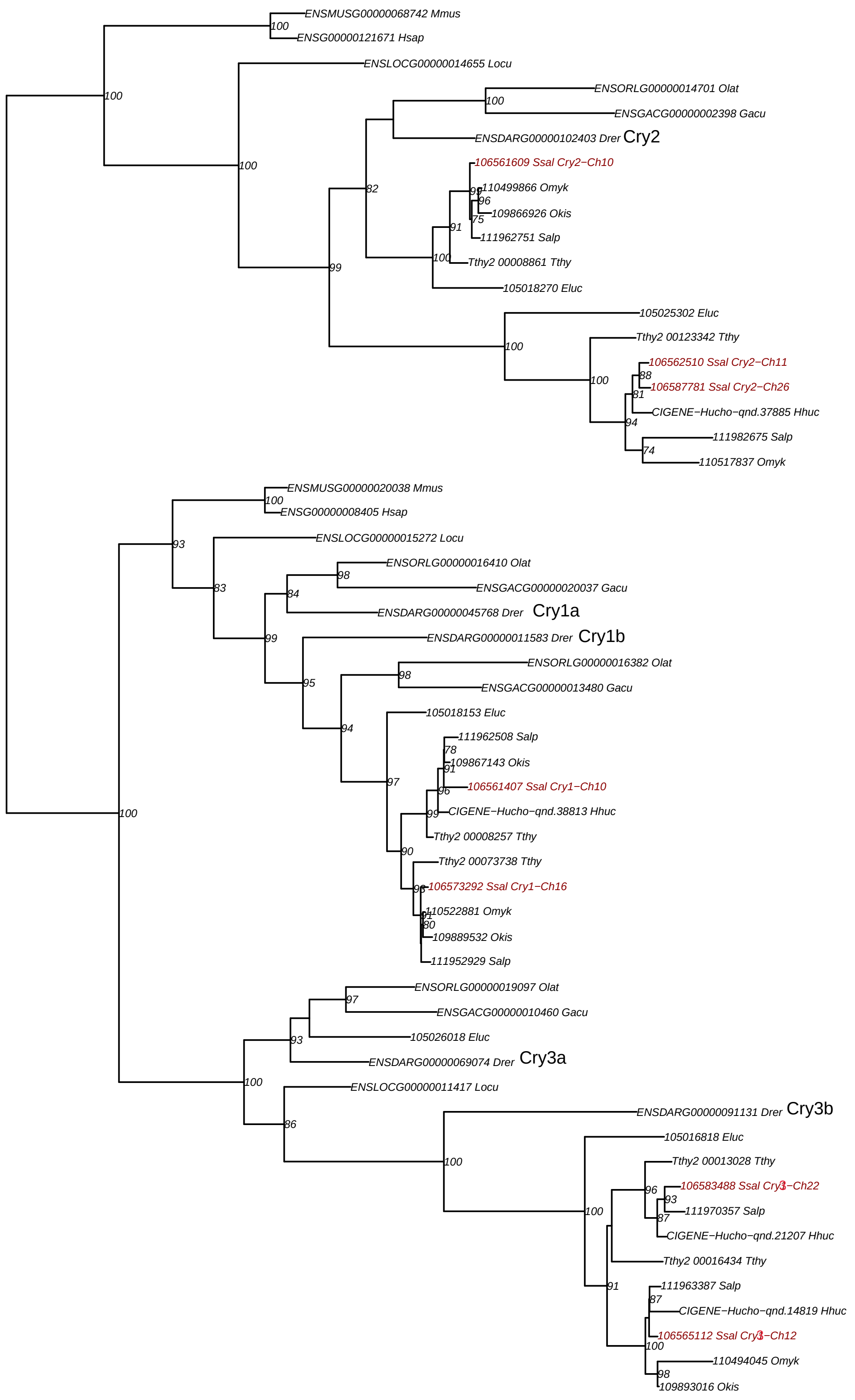

OG1v0000681 CLOCK/NPAS

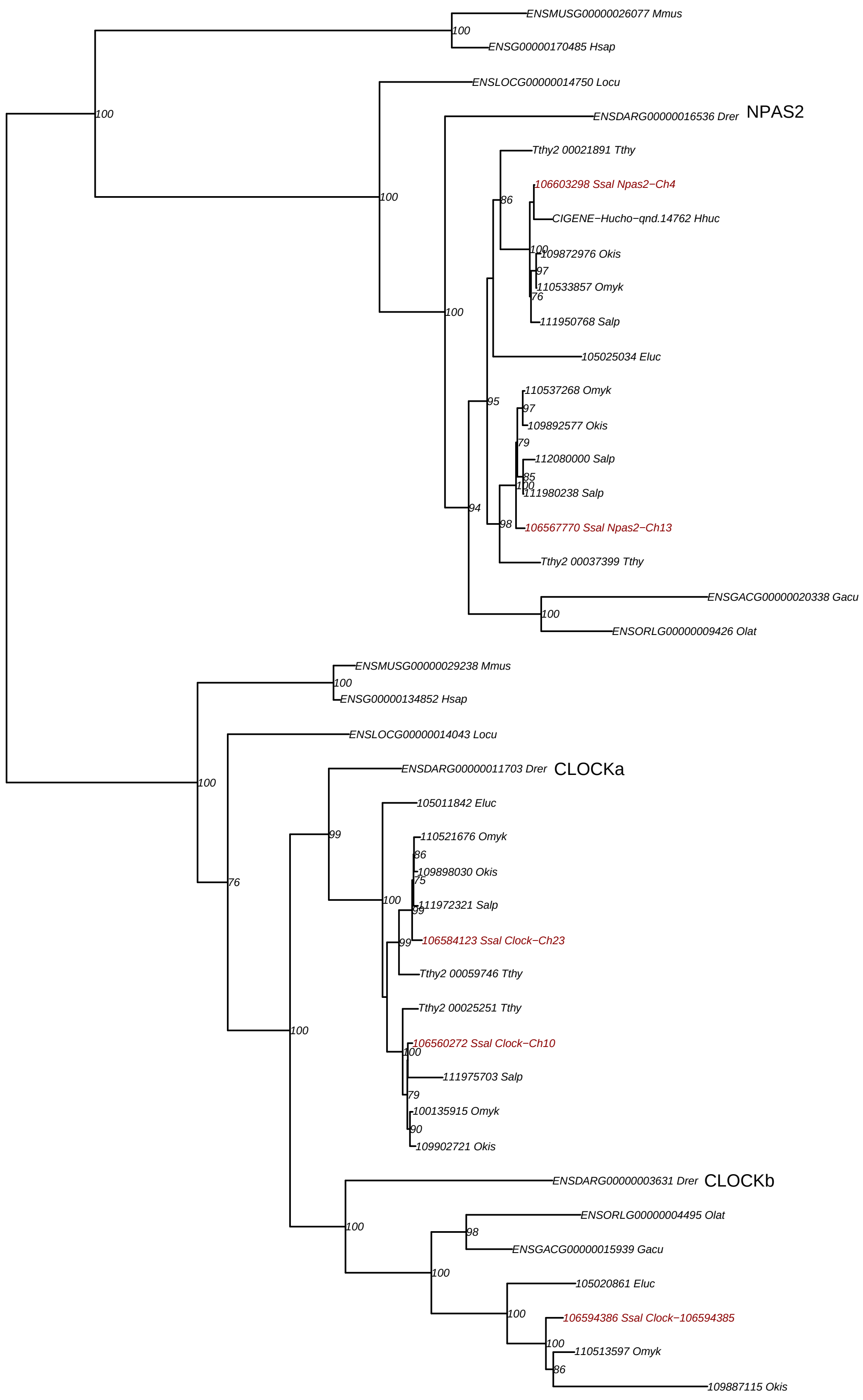

OG1v0001103 TEF

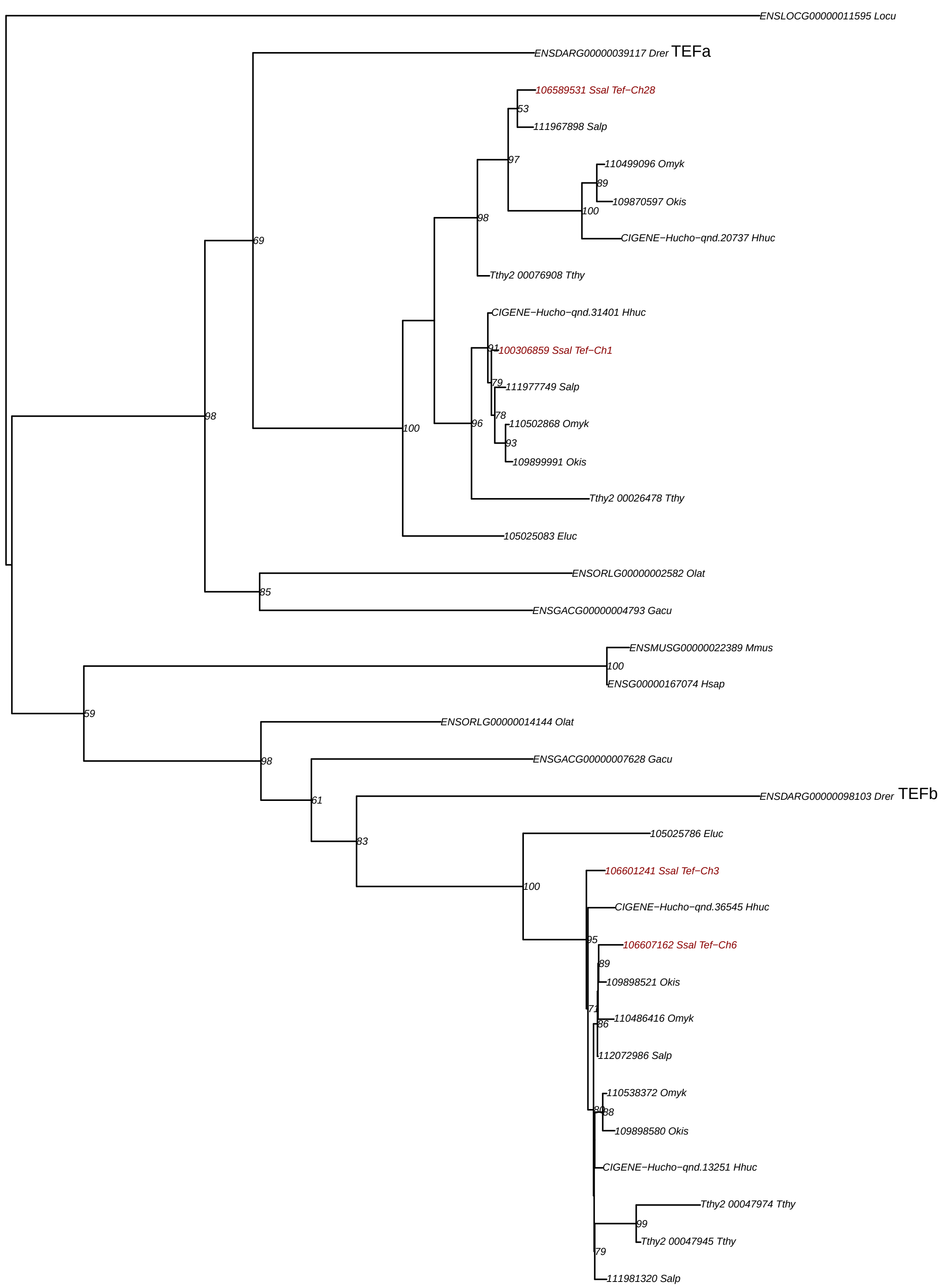

OG1v0001835 DBP

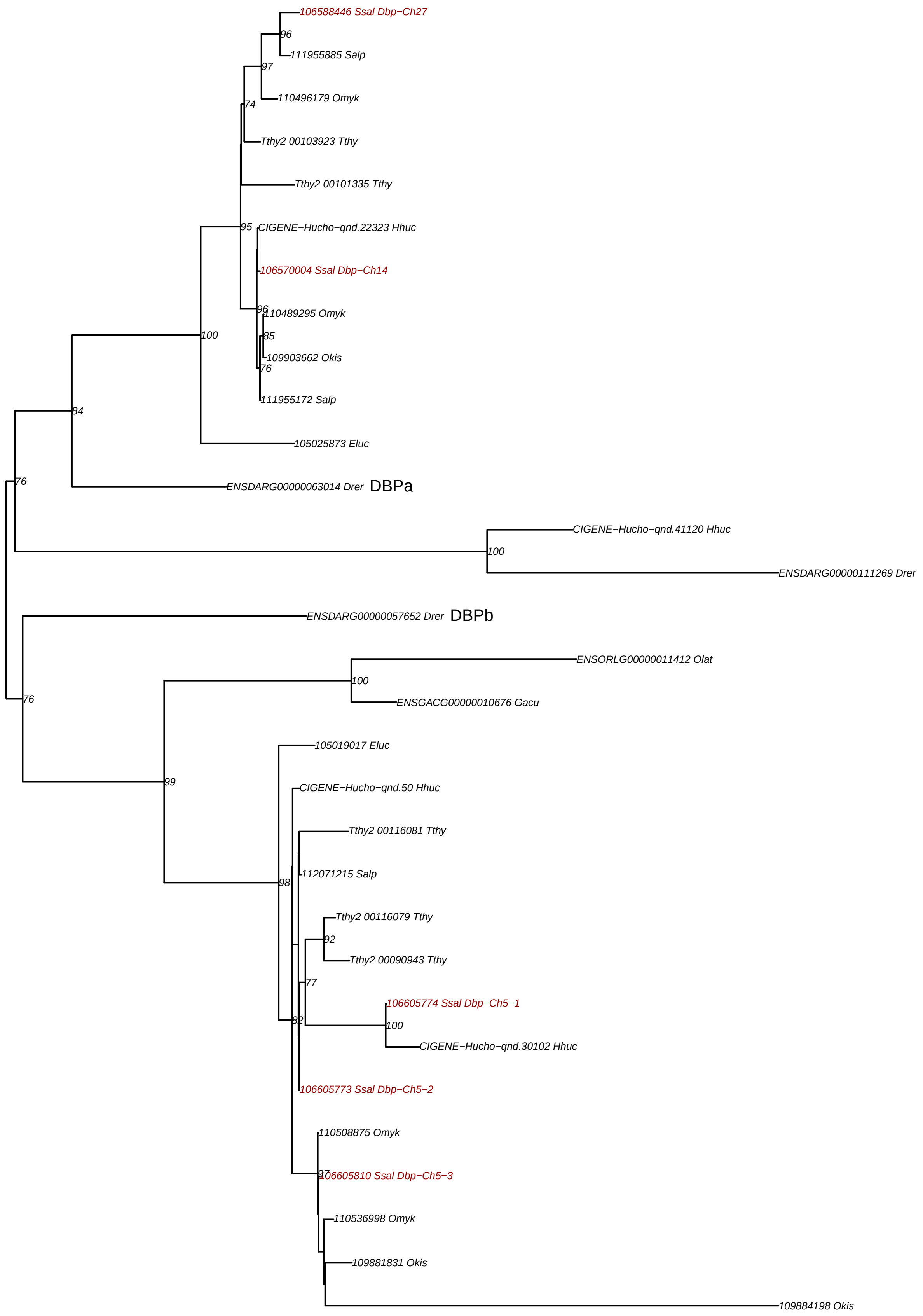

OG1v0002483 PER1

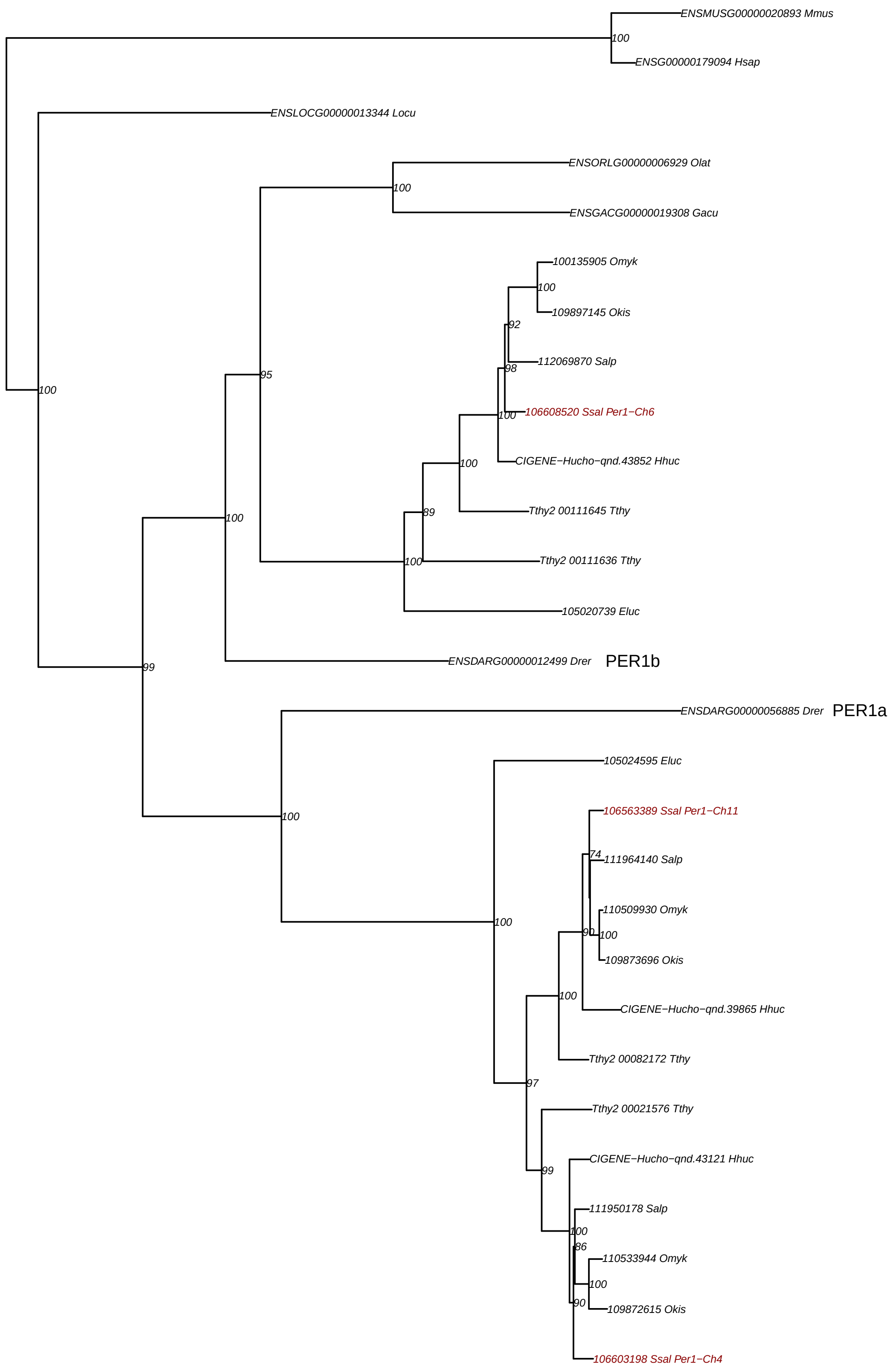

OG1v0002849 ARNTL1

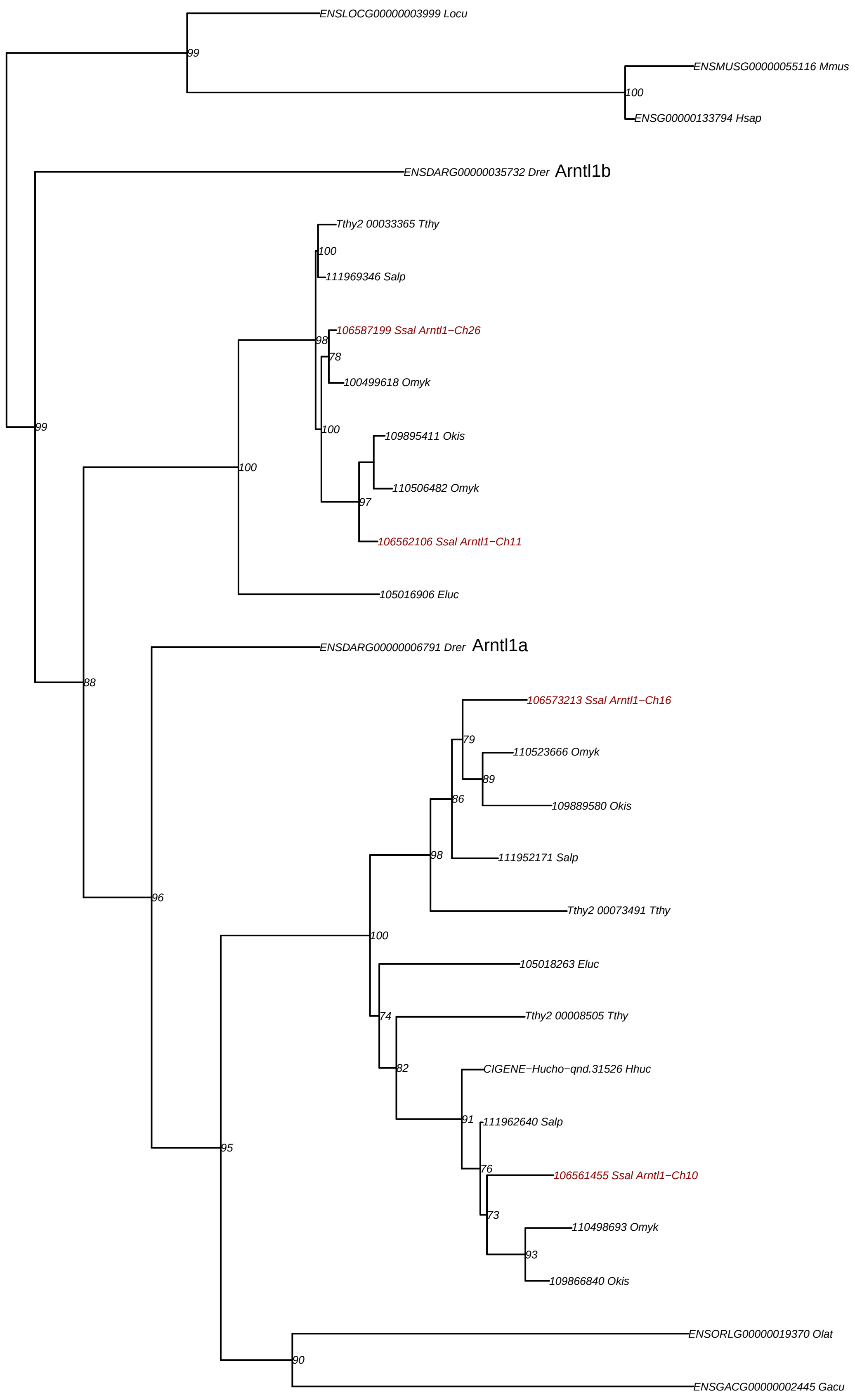

OG1v0003243 HLF

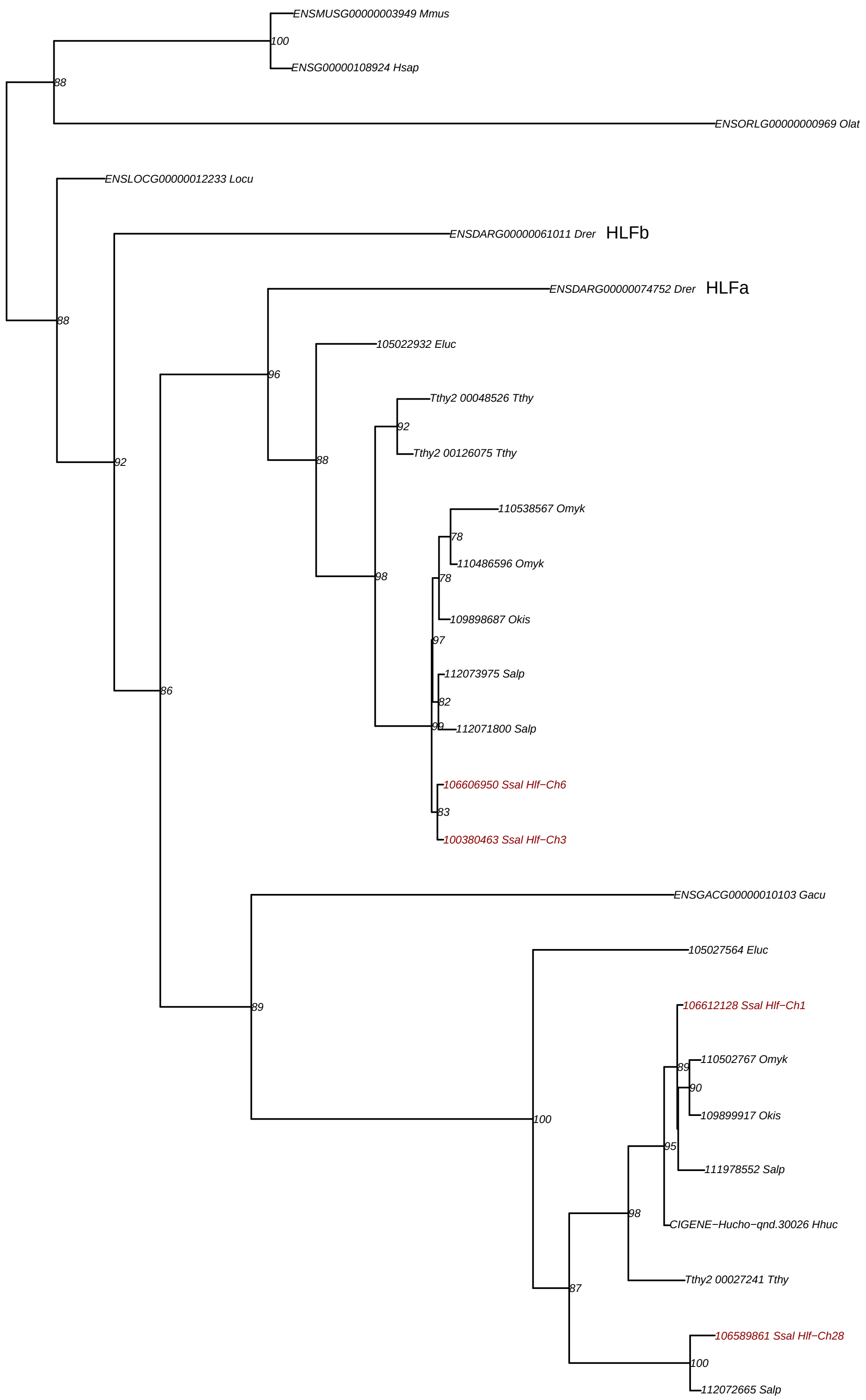

OG1v0003721 RORA

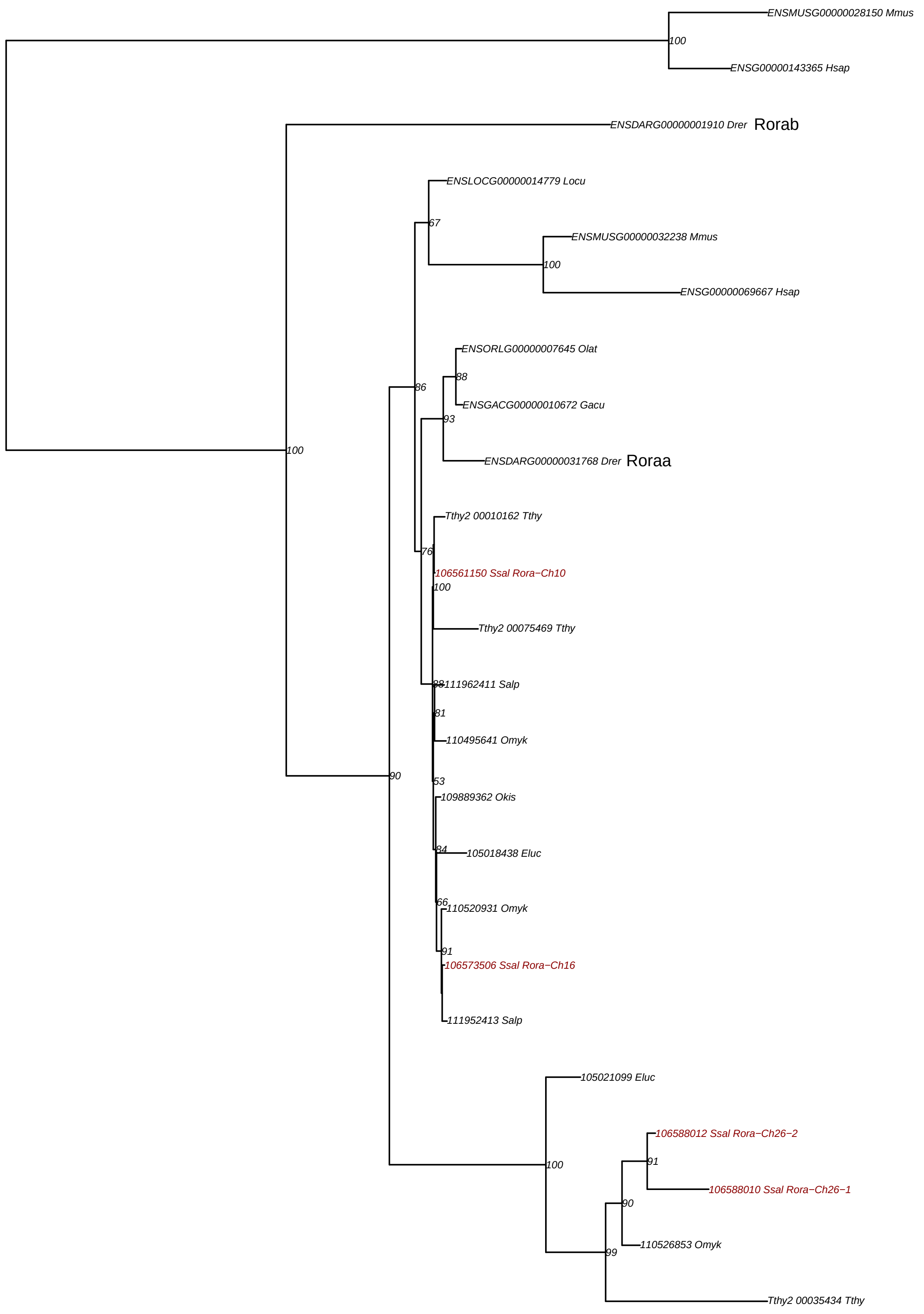

OG1v0003904 PER2

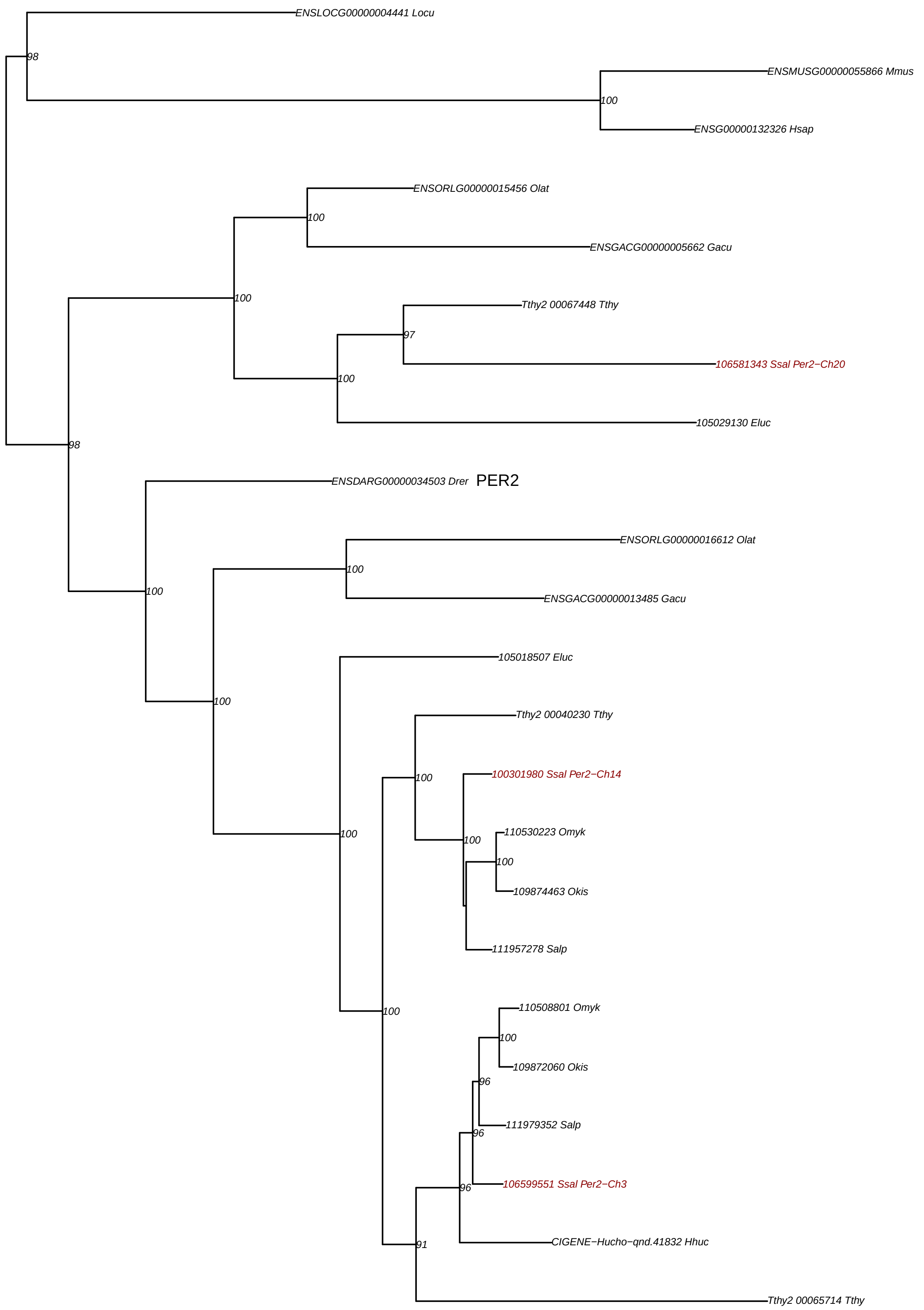

OG1v0007203 NR1D2b

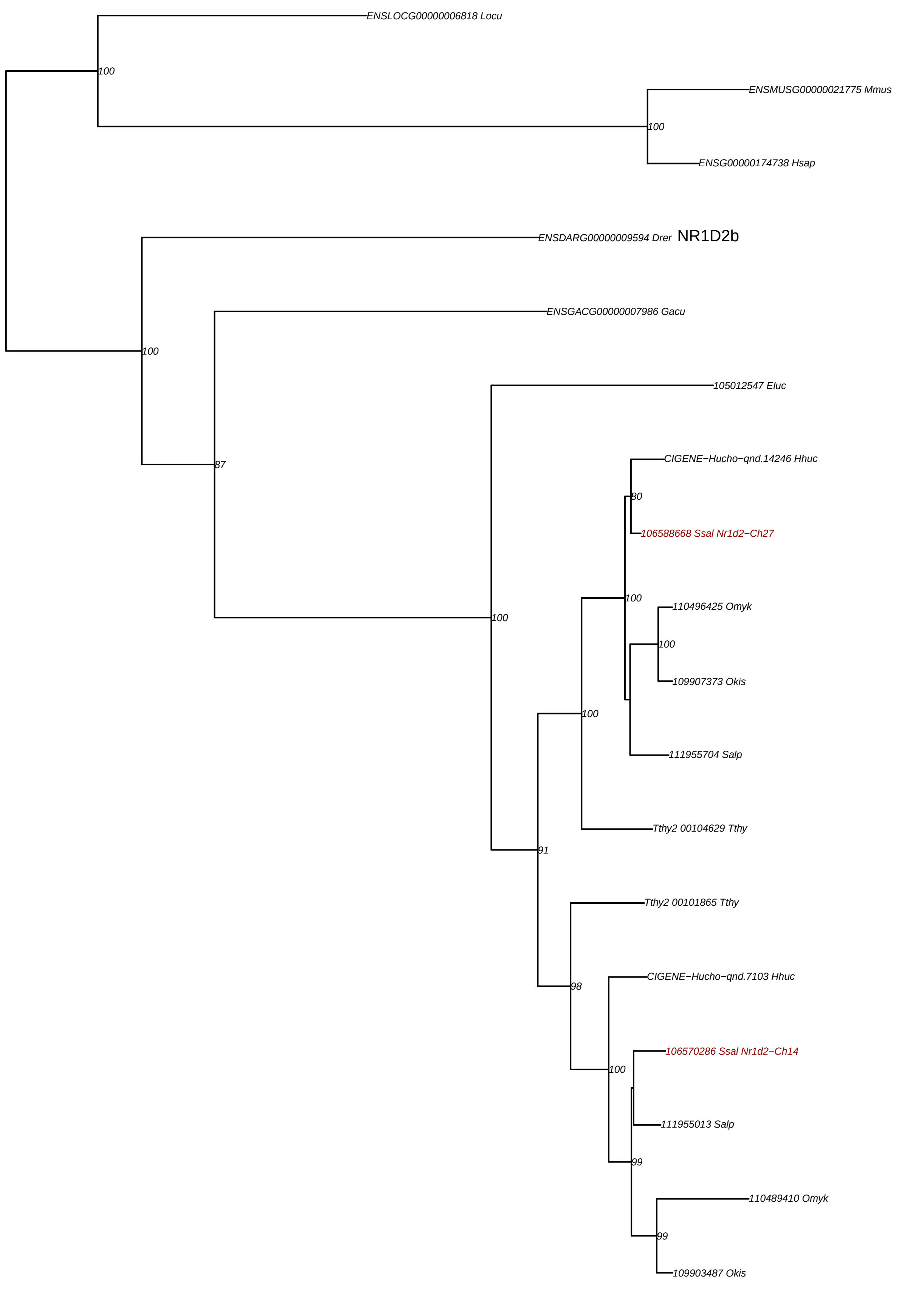

OG1v0007372 RORB

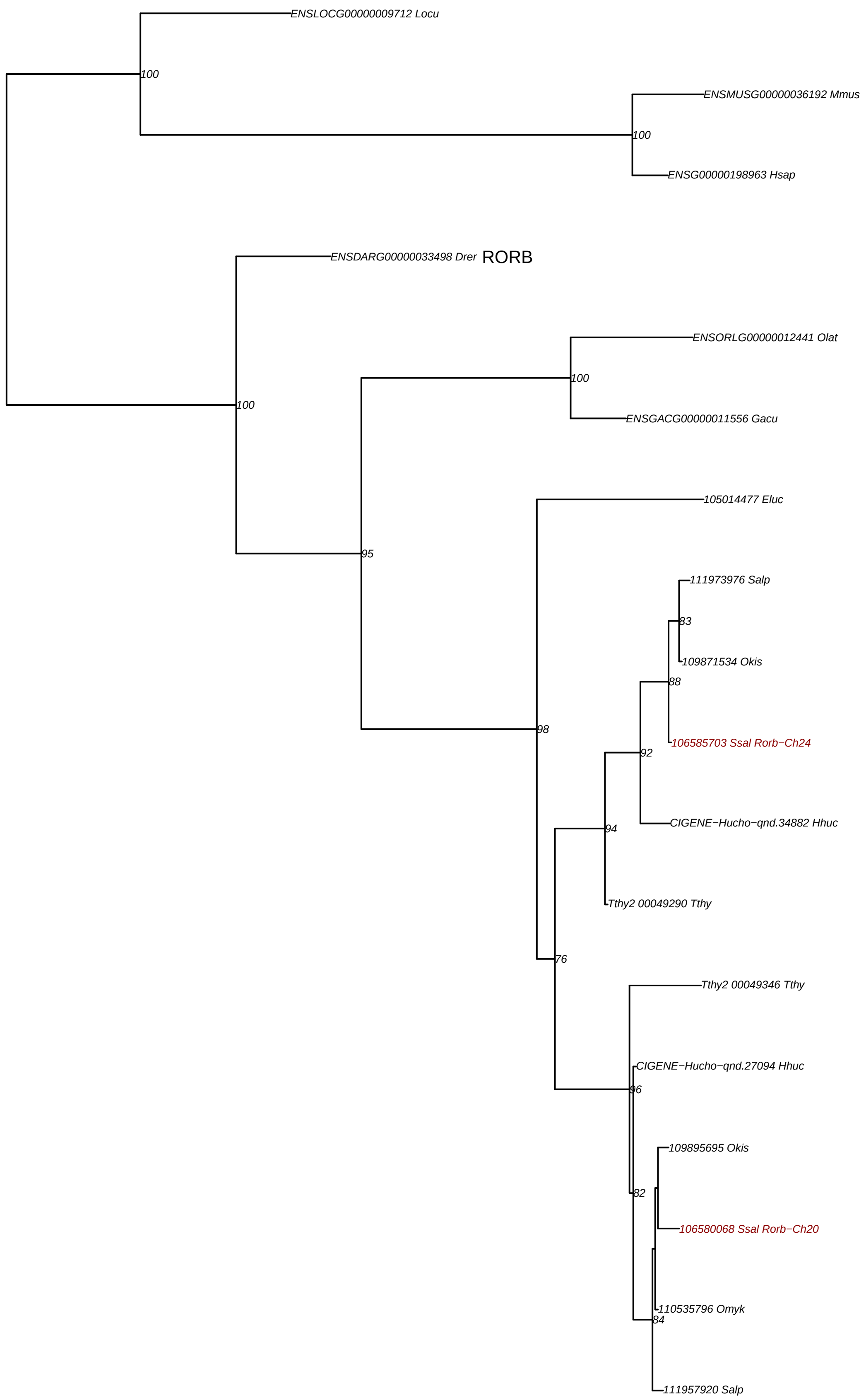

OG1v0007602 CIART

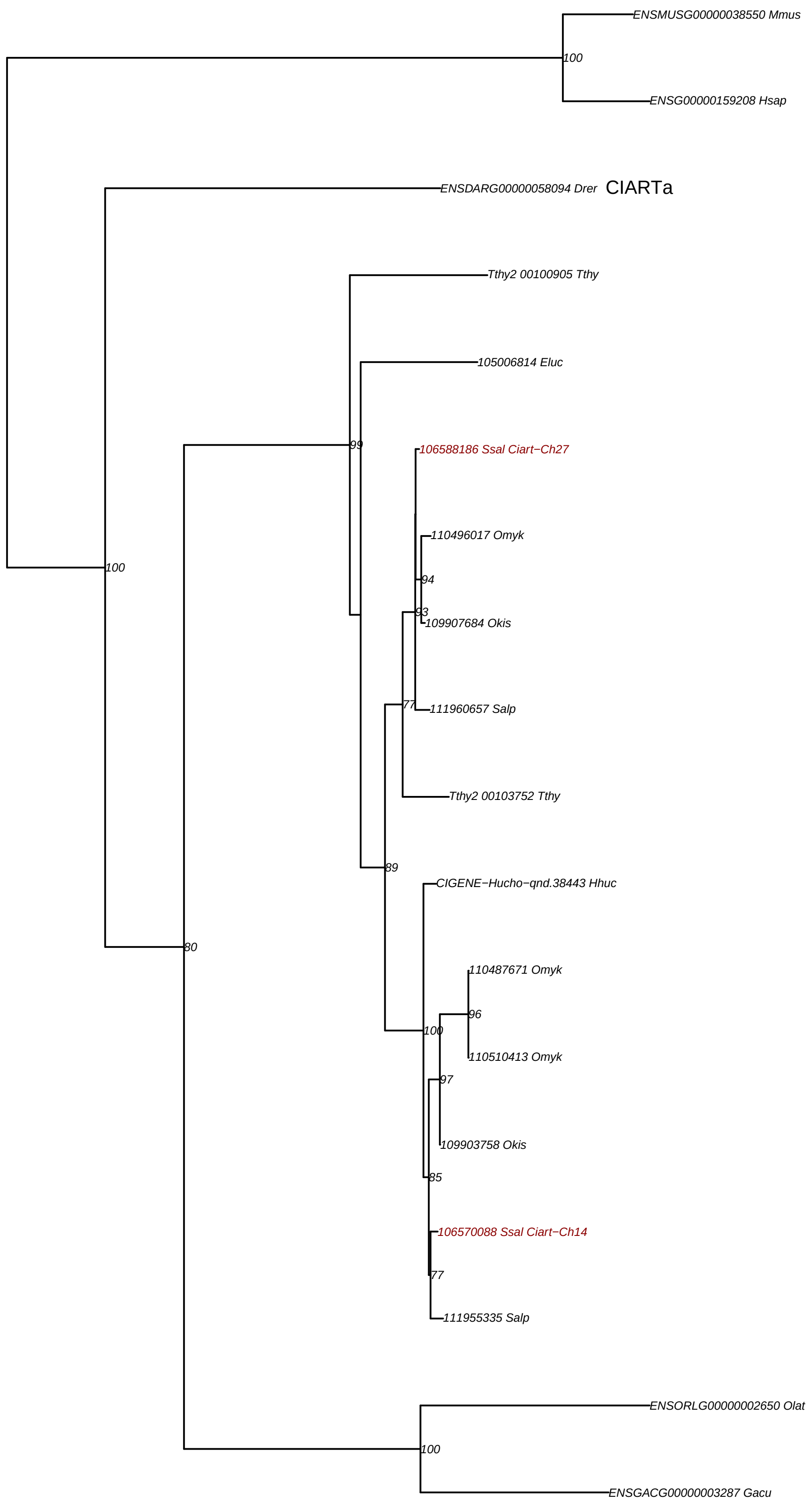

OG1v0010710 NR1D2a

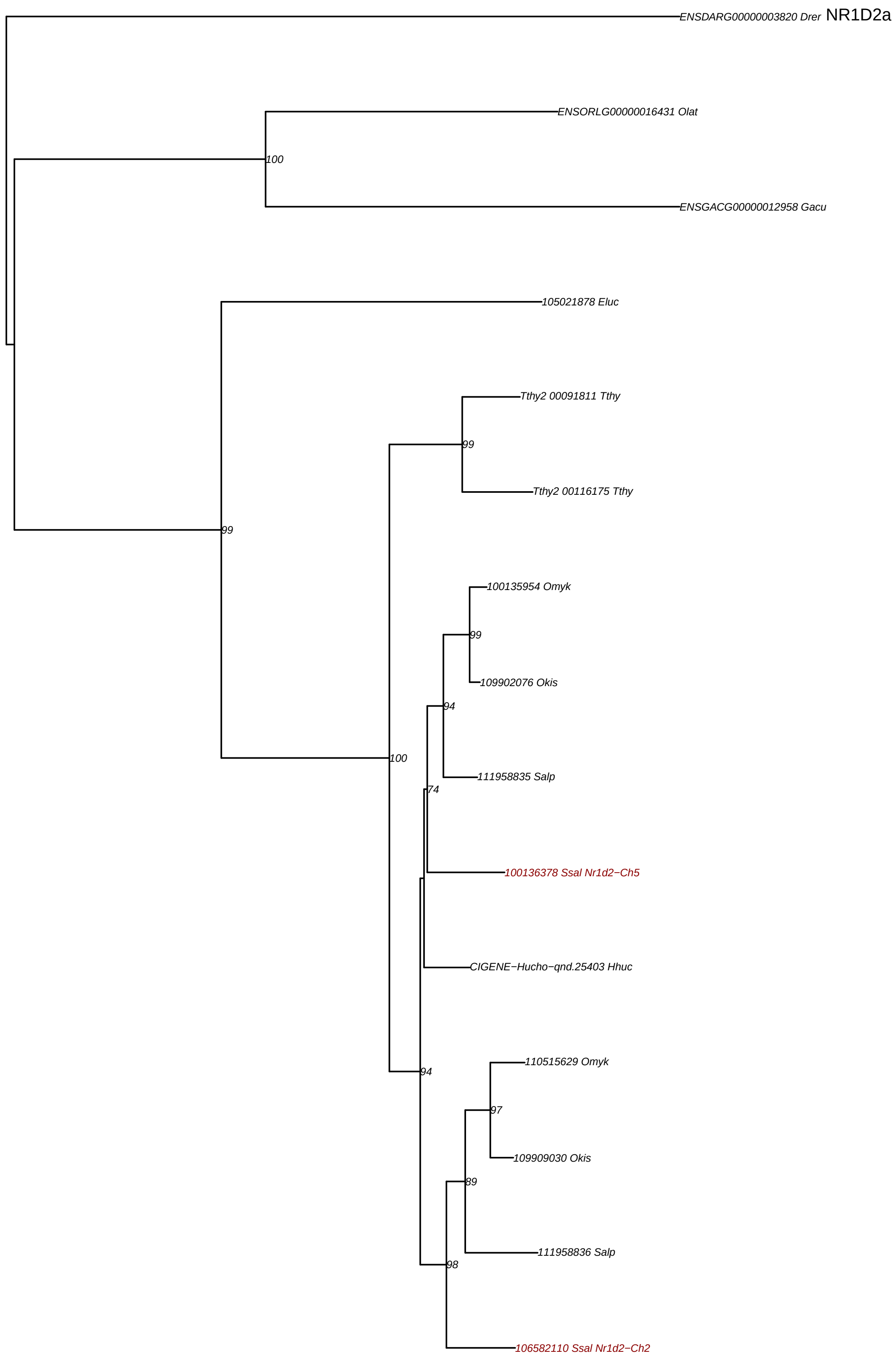

OG1v0011513 NR1D1

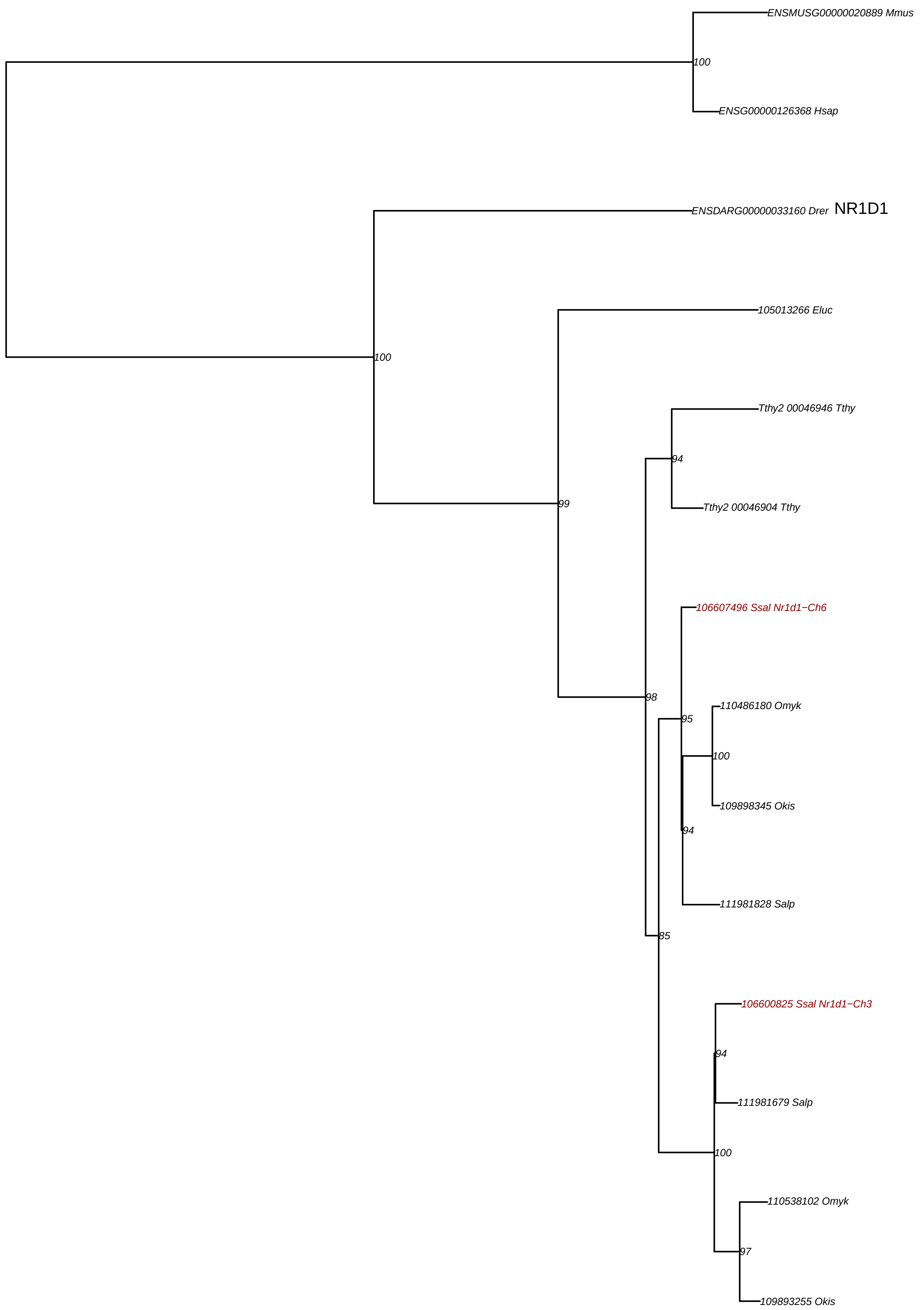

OG1v0013005 RORC

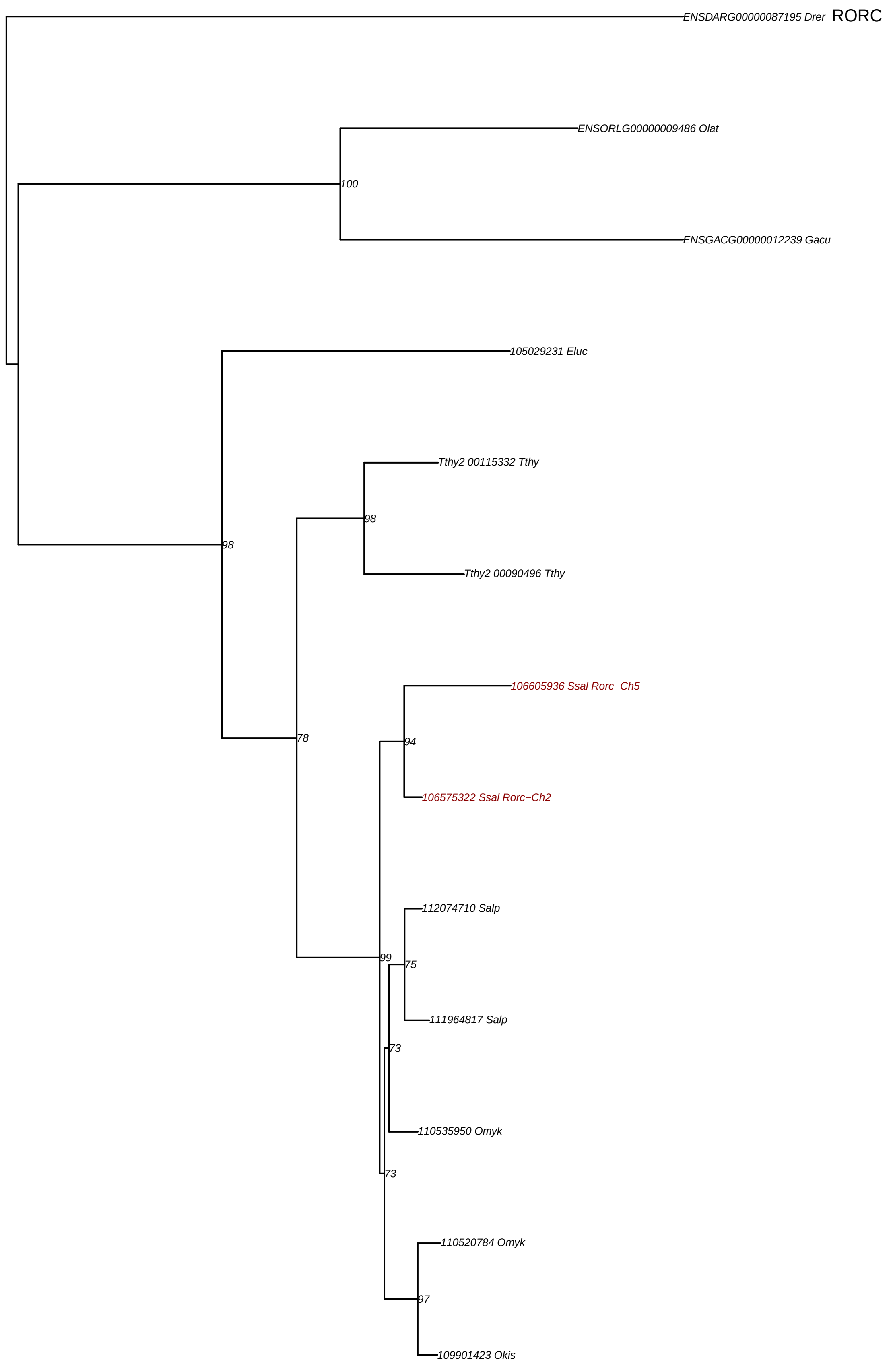

OG1v0015055 CRY

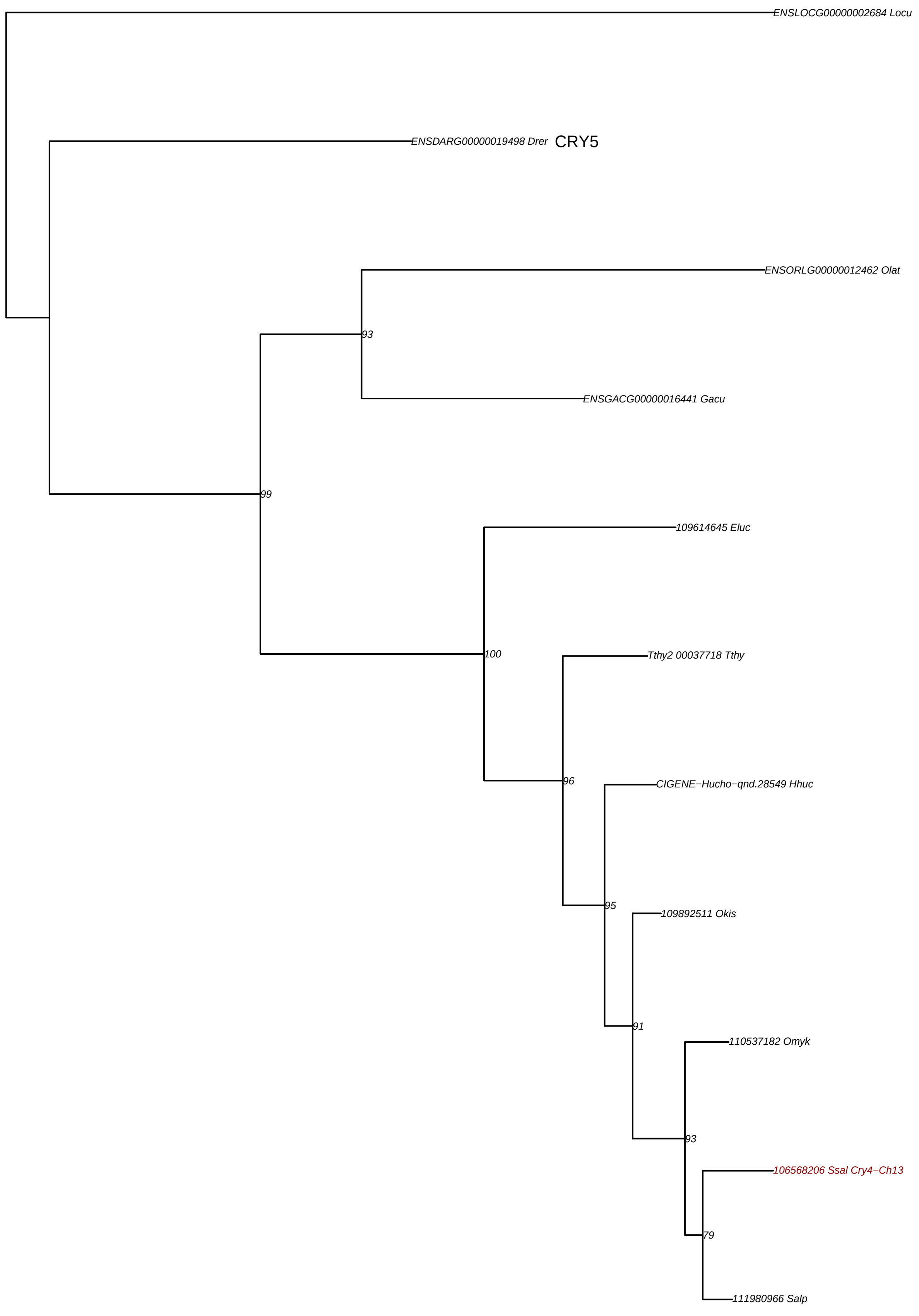

OG1v0016141 PER3

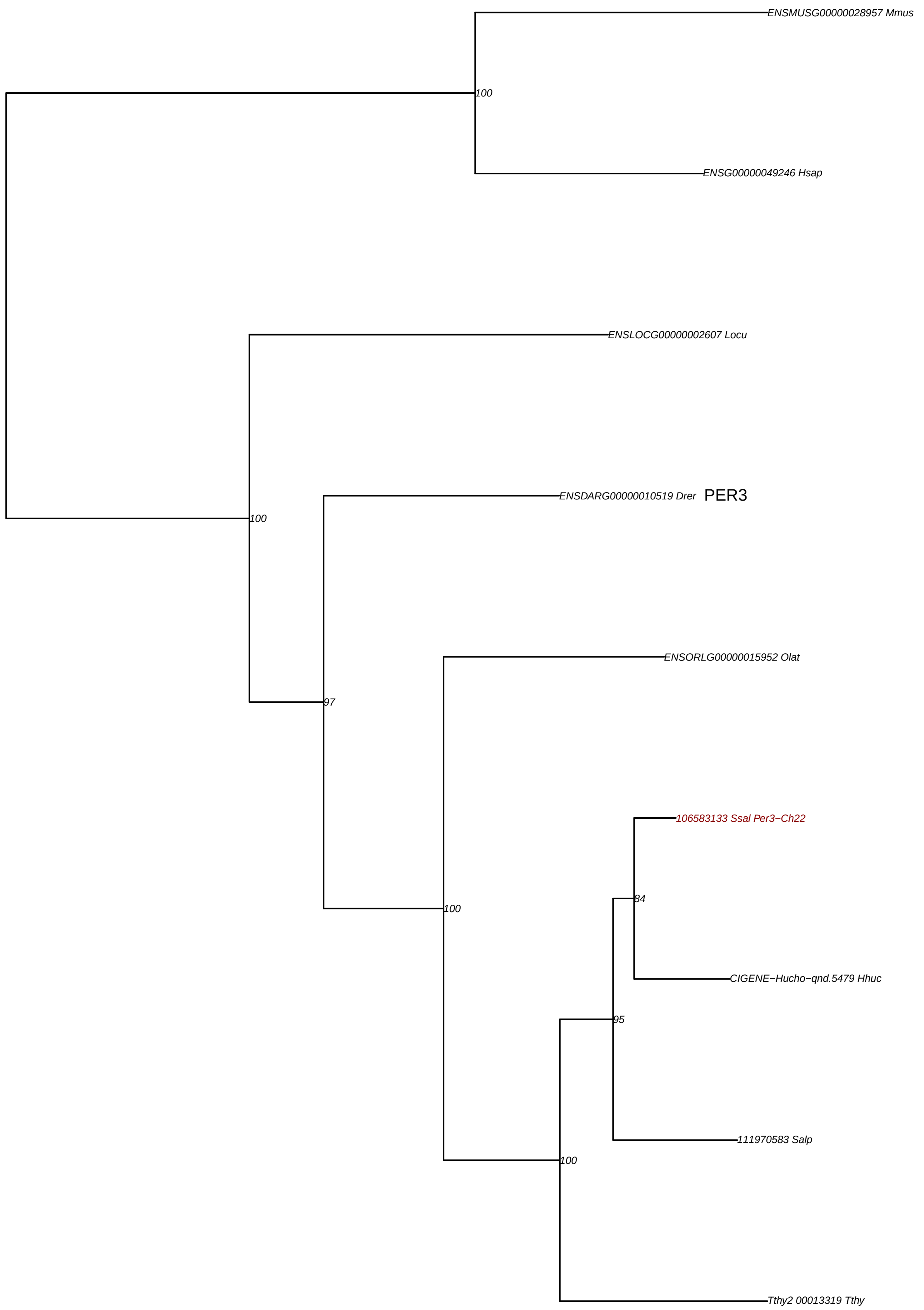

Supplement: S1 Appendix — (PDF) [file pgen.1009097.s011.pdf]
