# Supplementary material for: Diversified regulation of circadian clock gene expression following whole genome duplication
Source: PLoS Genet. 2020 Oct 8;16(10):e1009097. doi: 10.1371/journal.pgen.1009097 (PMC7575087; doi:10.1371/journal.pgen.1009097)
Supplement: Attachment — Submitted filename: Reviewers_comments.docx [file pgen.1009097.s013.docx]

This paper seems fundamentally experimentally sound, and the authors have generated a large amount of data, which I believe is interesting to both experts on salmonids and possibly circadian clocks. However, it lacks a significant conclusion and provides little novel data with no mechanistic insight. Although interesting for people working on salmonids, I question whether this is really interesting for a wider audience, and does it have sufficient importance to be published in Plos Genetics. I do not think so the way this paper is currently written, and I suggest publication in a specialty journal. The authors provide little in-depth analysis of their data, and below I reason why some of the conclusions or speculations made may not be correct.

Whole genome duplication of clock genes is indeed interesting, but I would believe that in order to make any functional predictions of the clock genes in salmonids, it would be natural to compare it to other teleost species. There is a broad historic literature on genome duplications in fish in general that is worthy of discussion. Teleost clocks, in both fresh and saltwater fish have been researched for over 20 years, by multiple groups. I also make several comments about this below.

I would not recommend publishing this paper in its current form.

**Major comments:**

Statement in the abstract: “Unexpectedly, we found a clear link between cortisol secretion and non-circadian changes in the expression of a subset of clock genes in the salmon gill.”

I would argue that the finding is somewhat expected (rather than unexpected), as in the field, we know that stress responses –do induce expression of some clock genes. If this has any biological meaning, such as here with the cortisol and stress, remains unanswered. The idea that clock genes can regulate downstream transcription in the absence of their oscillation is not novel and has been discussed in numerous mouse global transcriptome studies. Many clock genes (acting alone or as dimers) are simply transcriptional regulators and so will regulate gene expression, regardless of their clock function.

Statement in the abstract: “Overall, this analysis emphasises the potential for a richly diversified clock gene network to serve a mixture of circadian and non-circadian functions in vertebrate groups with complex genomes”

I am not sure this analysis does provide data for such speculation. As far as I am concerned this is just a lot of expression data, not proof of analysis that some genes act in a circadian way in one tissue, but not in another. My feelings are that although the data sets are very interesting, experimental manipulation would be required to determine the precise roles of these genes (in the clock mechanism or not). A lot of genes included in these two loops are not oscillating. Some are light responsive, some are not. This is already known. But the idea that each tissue might have a different (if subtle) clock mechanism is an interesting proposition. But how does one prove that? It would take an extensive and difficult set of molecular manipulations.

Three tissues were examined, and they do show a difference in expression. However, data from SV and OT is barely discussed at all. At no point do the authors ask or discuss the question “why or what genes are differentially regulated?” I believe interesting findings can be found here. It would be amazing if each tissue had a different clock mechanism, allowed as a consequence of these GWDs, but drawing this conclusion would at least require more sophisticated analysis of the gene expression profiles from each tissue, followed by a discussion of what the precise clock mechanisms might be in each tissue.

Neighbour joining-trees S1:
There is a lot of referencing of copies of clock genes to chromosomes in *Salmo salar*.

To a reader that does not have intimate knowledge of *S.salar* chromosomes (of which I believe there are few), cry1-Chr12 does not tell us anything about what kind of cryptochrome 1 it is and subsequently predict its likely function. I think this paper would benefit from neighbour joining trees showing the relationships to the clock genes of other fish which is better annotated with gene names rather than codes.

- Is there a reason the authors have not used annotated gene data from zebrafish and medaka? This would be natural to use as there has been some functionality studies on some of these genes in these fish.

- Is there a reason any values on the neighbour joining trees are not shown?

General comments for figure legends and/or methods: I cannot find any information in how many biological replicates were used in experiments, except from the seawater experiment. Please include this in any revision.

General comments to materials and methods/Figures/results:

-why did the authors choose a short photoperiod for all experiments? Why is the constant temperature variable from experiment to experiment? Will this impact gene expression patterns?

-I am not sure I understand how the experiment from Figure 3 was conducted. Did you sample across the day in juveniles or did you sample at the same time of day across multiple days? Did you sample on an LD cycle or in LL?

- line 197 no/weak oscillation in peripheral tissues as seen in 2B – it looks to me like the heatmap has some oscillating clock genes. Or am I interpreting the figure wrong?

- Did you collect blood samples pre or post smoltification, or during?

- In vitro gill culture: why have the authors not included a no-treatment control? DMSO can be toxic and can cause stress responses in cultures, depending on dose. A no-treatment control should be included to exclude effects from DMSO vs drug, or alternatively reference experiments where this has been done.

- The authors talk a lot about mammals vs salmon. I find the analysis a bit shallow and completely void of any general teleost clock knowledge. If you were to look into other fish species like zebrafish or medaka which has undergone one WGD less than the *S.salar,* you would find that actually, genes in clock loop1 are only slightly greater in number in *S.salar* compared to *D.rerio*. This may mean that functionality of the loop1 genes remains the same. The majority of the duplicated genes can actually be found in loop2, which in itself is an interesting observation, and I believe it should be discussed and analysed further. The idea that the different circadian loops might have more molecular and evolutionary “flexibility” is an interesting idea.

General comment to discussion. Differential regulation of clock genes in different tissues have been described previously in zebrafish though multiple microarray and RNAseq experiments and therefore this find cannot be described as novel. However, it is probably the first of its kind at this sampling frequency and in *Salmo salar*.

Line 265. So far, most fish examined are directly light sensitive and can set their local clocks. The role of an anatomical SCN is not clear, though there is a considerable literature on the role of the pineal gland in regulating locomotor activity and sleep. For example, blocking input from the pineal has an effect on some behavioural traits but not on local tissue clocks. These previous finds would mean that your speculations ‘that this shifting relationship between glucocorticoids and clock genes could be a contributory factor for the evolution of anadromy and the regulation of its seasonal timing’ would not hold up as the peripheral vs central clock organisation in teleostei are different and arose probably long before Ss4R, and earlier down the evolutionary tree of the teleostei.

Line 267-268 ‘Much of the genetic complexity seen in the salmonid circadian clockwork following the Ss4R event; we therefore asked how expression patterns diverged within Ss4R pairs.’ Again, how can you say this when you haven’t compared its complexity to other teleostei that haven’t undergone the Ss4R?

**Minor comments:**

Lines 52-55 and 55-56. These two sentences contain a lot of information and should be referenced.

Line 108: comma should be added after ‘duplicated regions’

Line 182 ss3r- I think the authors mean ss4r

Line 252 Comma after “Nonetheless”

Lines 236-237 Why are you not considering other circadian outputs such as cell cycle or immune systems?

Line 237-238 ‘Altered sensitivity to cortisol emerges as one proximate cause of differences in tissue-specific expression dynamics. The researchers haven’t looked at cortisol sensitivity in different tissues, if this is previous research it should be referenced.
