# Supplementary material for: Diversified regulation of circadian clock gene expression following whole genome duplication
Source: PLoS Genet. 2020 Oct 8;16(10):e1009097. doi: 10.1371/journal.pgen.1009097 (PMC7575087; doi:10.1371/journal.pgen.1009097)
Supplement: Attachment — Submitted filename: Reviewers_comments-response-06082020.docx [file pgen.1009097.s014.docx]

**Response to reviewers: PGENETICS-D-20-00531- Diversified regulation of circadian clock gene expression following whole genome duplication**

Reviewer comments are in *italics* and responses are in red.

We thank all reviewers for their comments and feel the manuscript is improved as a result. In addition to answering their comments we have made a number of edits to the text to improve readability and updated figures for clarity. All changes are indicated by the yellow highlight in the revised manuscript.

***Reviewer #1***

*This manuscript by West and colleagues tackles a central issue that has arisen in the study of circadian clock function in non-mammalian vertebrates, notably fish species. This concerns the functional significance of multiple copies of different types of clock gene that have arisen as the result of whole genome duplication. Does this reflect the presence of considerable functional redundancy or alternatively, do the multiple gene copies adopt different specialized functions and thereby confer even greater flexibility and adaptability on the circadian timing system during evolution? Until now, this issue has not been convincingly resolved. The authors of this manuscript approach the problem by studying a fascinating species, the Atlantic salmon, where multiple genome duplication events have lead to the extreme situation of there being 61 clock gene counterparts. Furthermore, the most recent genome duplication event has been attributed to enabling the evolution of anadromy a physiologically challenging adaptation whereby the fish spend parts of their life cycle in fresh water and other parts in seawater.*

*The authors charaterize daily changes in expression of these various clock genes in the gills, the optic tectum and the saccus vasculous as well as over the course of development. In particular, they compare the expression patterns of pairs of clock gene „ohnologues“. The results indicate strong gene and tissue-specific divergence in expression patterns consistent with functional diversification. Furthermore, a link between cortisol secretion and non-circadian expression of a subset of the clock genes is observed in the gill, which is thought to constitute part of the adaptation to the transition from fresh to seawater.*

*This represents a valuable and fascinating piece of work that will boost the relevance of many studies which explore circadian clock gene function and regulation in non-mammalian vertebrate models as well as providing valuable new insight for studying the evolution of the physiological function of cortisol. The data are well presented, convincing and are interpreted in a reasonable way*.

We thank the reviewer for their positive comments and are pleased they see the value of our work.

*I have one general point of criticism that concerns the use of purely mammalian (mouse-derived) data to guide the choice and interpretation of the clock genes under investigation. In particular, predominantly following the work and conclusions of Uli Schibler, the PAR domain transcription factors TEF, DBP and HLF are all considered as clock-related genes - serving as clock regulated factors that mediate feedback to the core clock loop mechanism. To my understanding, several publications have now shown that this view of PAR factor function is clearly not the case in fish. These transcription factors have been instead identified as mediating light and UV induced transcription of clock genes such as per2 and Cry1 as well as other types of gene involved in DNA damage repair. Interestingly, they also appear to serve as mediators of oxidative stress-induced gene expression (Pagano et al., Scientific reports 2018). I think the key point here is that D-box enhancers, which act as the regulatory targets of these PAR factors, are NOT clock regulated in fish. This important observation is completely ignored in the text and actually raises a more general point that would actually support the main conclusions of this manuscript, namely that clock gene function and regulation is inherently plastic during evolution, and so major changes in the role of particular sets of clock components may be entirely expected. The introduction and discussion sections should be adapted to include this missing relevant information.*

We agree that more information on zebrafish should be added to the manuscript and have updated the manuscript (see below for details). Further to this we have tried to make the data more interpretable in the context of zebrafish (and other species) by providing maximum likelihood gene trees with bootstrap values for all genes included in the study (S1 appendix). These gene trees are available in supplementary data and include the following species: human, mouse, spotted gar, zebrafish, stickleback, medaka, northern pike, grayling, danube salmon, arctic charr, coho salmon, rainbow trout, Atlantic salmon. We have updated supplementary table 1 to include the orthologues in zebrafish, medaka and mouse for each of the salmon genes to allow for easy cross comparison.

Page 5 line 88-90: “Of the 18 clock genes (as defined in Figure 1B) identified in laboratory mice the zebrafish genome contains 30, and the Atlantic salmon genome contains 61 clock genes (S1 Table, S1 appendix)”.

Page 7 line 104-106: “Comparing the repertoire of clock genes in spotted gar (2 WGDs), zebrafish (3 WGDs), and Atlantic salmon (4 WGDs) (Figure 1A) we find no difference in duplicate for loop 1 versus loop 2 genes (Figure 1B)”.

Page 7 line 110-113: “The S1 Table lists the specific gene identifiers and orthologues to zebrafish, medaka, spotted gar and mouse”.

Page 15-16 line 277-301 “Previous work has identified several Atlantic salmon clock genes that cycle in diel conditions [51–53]. While confirming these earlier findings, our analysis goes far beyond them by showing how the entire clock network is regulated under diel and constant conditions in serval tissues. We show that only the OT shows persistent rhythmicity under constant conditions, not the SV or gill (Figure 2). These results sit in contrast to the zebrafish circadian system in which tissue autonomous clock gene oscillation are seen in all tissues investigated [54]. Specific elements of the zebrafish clock appear to be fundamentally different to the Atlantic salmon clock. For example zebrafish Cry1a is acutely light-induced and appears to pause the circadian mechanism under LL [9], whereas the closest salmon orthologues to zebrafish Cry1a, Cry1-Ch10 and Cry1-Ch16, are not light-induced and show persistent rhythmicity in the OT under LL (S1 Table). Instead the salmon orthologues to zebrafish Cry3b, Cry3-Ch22 and Cry3-Ch12, are acutely induced by light and salt, respectively. Light induction Zebrafish Tefa is light-induced [33] this is not seen in the closest salmon orthologues Tef-Ch1 and Tef-Ch28, however, the zebrafish Tefb orthologues, Tef-Ch3 and Tef-Ch6 are seawater induced. In zebrafish, Tef transcription is induced in response to light and UV exposure producing reactive oxygen species (ROS) which act on the D-box in the Tef promoter, initiating a DNA damage repair cascade [55]. Importantly this light-mediated ROS inducibility of Tef appears to be absent in cave fish and mammals [55], suggesting a zebrafish-specific adaptation to UV light exposure, potentially related to spawning environment [56]. In our study we found no enrichment for genes associated with oxidative stress in either the seawater challenge or photoperiodic treatment groups but the photoperiod driven smoltification induction of all four Tef genes is noteworthy, as is the seawater specific induction of the ss4r pair Tef-Ch3 and Ch6, and the apparent regulatory divergence of these duplicates in glucocorticoid responsiveness. These species differences indicate plasticity of the circadian clock in response to different environmental stressors depending on life history”.

*A related point: I am not sure about the current understanding of the mechanisms involved in the osmotic stress response of the gills, but might the observed differential regulation of TEF genes during the response to osmotic stress support some role for oxidative stress in this process?.....*

We thank the reviewer for this interesting suggestion. However, we find no evidence of an over-representation of genes associated with oxidative stress in either our seawater challenge or photoperiodic RNAseq datasets. Please also see our updated discussion regarding tef (see above and in manuscript Page 15-16 line 277-301).

*Other points emerging from the text:*

*Line 53: What do the authors mean by (co-)activator and (co-)repressor? There are transcription factors that directly bind to DNA sequences and co factors that interact with transcription factors and other parts of the transcription machinery but do not directly bind to DNA. So, it would be more accurate to mention transcription factors and cofactors or coregulators.*

Thank you for the suggestion we have clarified the text accordingly:

Page 4 line 51-54: “In eukaryotes, circadian control derives from a cell-autonomous molecular oscillator, assembled from a regulatory network of transcription factors, co-factors, (co-)regulators, chromatin modifiers and an array of post-translational regulators of protein function, often described collectively as ‘clock genes’ [1].”

*Line 131 and 147: Describing the optic tectum as „highly light-responsive“ at this point in the text is a little misleading - specifically because of the existence of directly light responsive peripheral tissues in fish. This is more clearly explained later in the discussion (retinal input, expression of photoreceptors etc.), but I think to avoid confusion, it would make more sense to also clarify this point in the introduction section.*

We have updated the text to reflect the reviewers comment

Page 7 line 131: “….processing and is coupled directly and indirectly to light input (1–4)”,

Page 8, line 147: “….Hence robust circadian rhythmicity is a feature of the salmon brain, but gene expression rhythms are severely dampened in the peripheral tissues we studied”.

The direct light responsiveness of peripheral tissues in fish is almost exclusively based on zebrafish, therefore we feel it is misleading to make reference to this in the introduction as a general fish feature. Our data also do not support this, both the SV and gill do not show direct light responsiveness. We have updated our discussion to clarify this point and discuss the differences between salmon and zebrafish. Please see above and in manuscript Page 15-16 line 277-301.

*Line 217: „during in stress“ should be corrected to „during stress“.*

This has been corrected.

***Reviewer #2***

*This paper seems fundamentally experimentally sound, and the authors have generated a large amount of data, which I believe is interesting to both experts on salmonids and possibly circadian clocks. However, it lacks a significant conclusion and provides little novel data with no mechanistic insight. Although interesting for people working on salmonids, I question whether this is really interesting for a wider audience, and does it have sufficient importance to be published in Plos Genetics. I do not think so the way this paper is currently written, and I suggest publication in a specialty journal. The authors provide little in-depth analysis of their data, and below I reason why some of the conclusions or speculations made may not be correct.*

*Whole genome duplication of clock genes is indeed interesting, but I would believe that in order to make any functional predictions of the clock genes in salmonids, it would be natural to compare it to other teleost species. There is a broad historic literature on genome duplications in fish in general that is worthy of discussion. Teleost clocks, in both fresh and saltwater fish have been researched for over 20 years, by multiple groups. I also make several comments about this below.*

*I would not recommend publishing this paper in its current form.*

We are glad the reviewer feels our study is well conducted and interesting to a variety of researchers. Regarding the reviewers comments on the suitability of our work for plos genetics we do feel that our work is of broad enough interest and is relevant for chronobiology, evolutionary genomics, evolutionary-development, endocrinology and fish physiology. However, we have endeavoured to improve the manuscript though additions of information relevant to other teleosts and specifically paid attention to the zebrafish literature which constitutes the best conducted chronobiology research in a fish.

Specifically we have added maximum likelihood gene trees with bootstrap values for all genes included in the study (S1 appendix). These gene trees are available in supplementary data and include the following species: human, mouse, spotted gar, zebrafish, stickleback, medaka, northern pike, grayling, danube salmon, arctic charr, coho salmon, rainbow trout, Atlantic salmon. We have updated supplementary table 1 to include the orthologues in zebrafish, medaka and mouse for each of the salmon genes to allow for easy cross comparison.

We have updated the manuscript in several places, please see response to reviewer 1 above and Page 5 line 88-90, Page 7 line 104-106, Page 7 line 110-113, Page 15-16 line 277-301

We agree that it is natural to make contrasts to other telosts and have endeavoured to do so, to truly make definitive functional predictions we would need to make contrasts to expression data in closely related outgroups i.e. Esox, this would of course be very interesting and has potential to shed light on the apparently diverse relationships to biological timekeeping we seem to observe in zebrafish versus salmon but we feel it is beyond the scope of a single study.

**Major comments:**

*Statement in the abstract: “Unexpectedly, we found a clear link between cortisol secretion and non-circadian changes in the expression of a subset of clock genes in the salmon gill.”*

*I would argue that the finding is somewhat expected (rather than unexpected), as in the field, we know that stress responses –do induce expression of some clock genes. If this has any biological meaning, such as here with the cortisol and stress, remains unanswered. The idea that clock genes can regulate downstream transcription in the absence of their oscillation is not novel and has been discussed in numerous mouse global transcriptome studies. Many clock genes (acting alone or as dimers) are simply transcriptional regulators and so will regulate gene expression, regardless of their clock function.*

The unexpected nature of this result refers to the non-circadian (and non-stress) changes in clock gene expression. In mammals there is a very clear link between rhythmicity of cortisol expression and clock gene rhythmicity – this is absent here. Using a well-known photoperiodic paradigm to induce smoltification which is associated with an increase in cortisol over weeks (not acutely and not a stress response) we see increases in clock gene expression – this is surprising as the effects the reviewer describes are all based on short term/acute stress induction experiments. We will concede to removing the word “unexpectedly” but we do believe this is a novel finding in relating to a specific tissue function and a seasonal adaptation.

*Statement in the abstract: “Overall, this analysis emphasises the potential for a richly diversified clock gene network to serve a mixture of circadian and non-circadian functions in vertebrate groups with complex genomes”*

*I am not sure this analysis does provide data for such speculation. As far as I am concerned this is just a lot of expression data, not proof of analysis that some genes act in a circadian way in one tissue, but not in another. My feelings are that although the data sets are very interesting, experimental manipulation would be required to determine the precise roles of these genes (in the clock mechanism or not). A lot of genes included in these two loops are not oscillating. Some are light responsive, some are not. This is already known. But the idea that each tissue might have a different (if subtle) clock mechanism is an interesting proposition. But how does one prove that? It would take an extensive and difficult set of molecular manipulations.*

We are glad to see the reviewer acknowledge that ‘the data sets are very interesting’. Yes, to truly nail the question of circadian VS non-circadian regulation at a mechanistic level an extensive amount of molecular work (likely years worth) is required, and is far outside the scope of this manuscript. Even if ‘expression data’ is not the ultimate proof of mechanism, the use of expression data to find “when” and “where” genes are expressed is a first and necessary step towards a mechanistic insights of circadian regulation, and functional diversification (e.g. see Carroll SB. Evo-Devo and an Expanding Evolutionary Synthesis: A Genetic Theory of Morphological Evolution. Cell. 2008;134: 25–36. doi:10.1016/j.cell.2008.06.030 & Carroll SB. Endless Forms. Cell. 2000;101: 577–580. doi:10.1016/S0092-8674(00)80868-5). With reference to our speculation – we tried not to make a definitive statement by using the words “emphasises” and “potentially”, and have not attempted to mislead the reader by making definitive statements.

*Three tissues were examined, and they do show a difference in expression. However, data from SV and OT is barely discussed at all. At no point do the authors ask or discuss the question “why or what genes are differentially regulated?” I believe interesting findings can be found here. It would be amazing if each tissue had a different clock mechanism, allowed as a consequence of these GWDs, but drawing this conclusion would at least require more sophisticated analysis of the gene expression profiles from each tissue, followed by a discussion of what the precise clock mechanisms might be in each tissue.*

We agree with the reviewer that more analysis can be conducted into the tissue specific differences between the OT, SV and gill. We provide a comparison of the circadian dynamics between these tissues, and note that only the OT seems to be oscillating, all data are available for other researchers to unpick, we did not think a gene by gene discussion of the OT, SV and gill would be appropriate given the other large amounts of data to discuss. Our primary point was that oscillations are dampened and may be functionally absent in tissues other than the OT – which leads us to our investigations in the gill on the expression of clock genes which are seemingly driven by season and seawater. A discussion of the *precise* clock mechanism in each tissue would be speculative without further experiments (see comment above). However, in the discussion lines 268 - 276 we do discuss the tissue differences: “Given these differences in tissue function, it is not surprising to observe different complements of clock genes in the two tissues and dramatic differences in temporal dynamics (Figure 2). In the diel-sensitive OT a dominant diel / circadian influence on expression is seen, while in the gill this is a much weaker influence. Nonetheless, clock gene expression in the gill is dynamically regulated, both over the chronic developmental time-scales of smoltification, and acutely in response to osmotic stress. The finding that the complement of genes showing highly sensitive changes in expression in the gill is largely non-overlapping with that under light / circadian control in the OT is clear evidence for diverged expression regulation within the circadian network”.

*Neighbour joining-trees S1:*

*There is a lot of referencing of copies of clock genes to chromosomes in Salmo salar.*

*To a reader that does not have intimate knowledge of S.salar chromosomes (of which I believe there are few), cry1-Chr12 does not tell us anything about what kind of cryptochrome 1 it is and subsequently predict its likely function. I think this paper would benefit from neighbour joining trees showing the relationships to the clock genes of other fish which is better annotated with gene names rather than codes.*

*- Is there a reason the authors have not used annotated gene data from zebrafish and medaka? This would be natural to use as there has been some functionality studies on some of these genes in these fish.*

*- Is there a reason any values on the neighbour joining trees are not shown?*

We agree with reviewer 2 that a more thorough and systematic presentation of the orthology/paralogy relationships between salmon genes and other teleosts/vertebrates would be helpful. In the new version of the paper we have therefore included maximum likelihood gene trees with bootstrap values (statistical node support) for all circadian pathway genes discussed in this manuscript (S1 appendix). These gene trees include the following species: human, mouse, spotted gar, zebrafish, stickleback, medaka, northern pike, grayling, danube salmon, arctic charr, coho salmon, rainbow trout, Atlantic salmon. We have updated supplementary table 1 to include the orthologues in zebrafish, medaka and mouse for each of the salmon genes to allow for easy cross comparison. Furthermore we have included more information in the methods section and added a link to the scripts and input data used in this analysis (<https://gitlab.com/sandve-lab/salmon-clockgenes/-/tree/master/>).

Page 18 line 345-3451: “Maximum likelihood gene trees with bootstrap values for all genes included in the study are presented in S1 appendix. These trees include the following species: human, mouse, spotted gar, zebrafish, stickleback, medaka, northern pike, grayling, danube salmon, arctic charr, coho salmon, rainbow trout, Atlantic salmon. The relevant scripts, input files and resulting trees are also available [here](https://gitlab.com/sandve-lab/salmon-clockgenes/-/tree/master/). For quick reference S1 table presents the salmon gene loci and their corresponding orthologues in Zebrafish, spotted gar, medaka and mouse”.

We agree that it is not common practice to use chromosome identifiers for Atlantic salmon gene names. We considered following more traditional gene nomenclature however we found that in dealing with duplicates this system quickly becomes difficult to follow – i.e. Nr1d2ba vs Nr1d2bb is more difficult to quickly scan and interpret compared to Nr1d2-Ch14 vs Nr1d2-Ch27. Therefore, we would like to maintain our current gene naming system. In order to clarify to the reader we have added the following explanation and updated S1 table:

Page 7 line 110-113: “ The chromosomal locations of the Ss4R pairs are shown on Figure 1C, and for ease of comparison the genes will be referred to by their gene name and chromosome. The S1 Table lists the specific gene identifiers and orthologues to zebrafish, medaka, spotted gar and mouse”.”.

We have also added a comparison to zebrafish in the results and discussion, please see response to reviewer 1 above and Page 5 line 88-90, Page 7 line 104-106, Page 7 line 110-113, Page 15-16 line 277-301 in the manuscript.

*General comments for figure legends and/or methods: I cannot find any information in how many biological replicates were used in experiments, except from the seawater experiment. Please include this in any revision.*

We apologise for the omission and have extensively up dated the manuscript methods and legends to reflect this information and generally improve the descriptions of the experiments performed:

Page 19 – 20 line 368 – 400: “

Circadian experiment I

Fish were maintained in 500L freshwater were transferred from LL to a short photoperiod (SP; 6L:18D) light schedule for 8 weeks before the start of the experiment. Short photoperiods provide a strong zeitgeber (timing signal) entraining the fish to a light dark cycle. In order to test for a circadian rhythm it is necessary to remove any zeitgebers, light, temperature and food are all potential zeitgebers which we controlled for. Two weeks before sampling, fish were distributed to two separate 150L tanks and water temperature was maintained at 14oC (±0.5). Fish were fasted for 48 hours prior to the experiment and throughout the sampling. To identify genes which were driven by an endogenous circadian rhythm we sampled under diel (SP; 6L:18D), constant light (LL) and constant dark conditions (DD). Sampling was at 4 hour resolution starting at ZT5 (zeitgeber time; time since lights on) in diel conditions (n= 3 per time point, 7 time-points), transitioning onto either LL or DD. In LL sampling was from CT9 (circadian time) at a 4 hour resolution (n = 3 per time-point, 6 time-points) because ZT 1 and 5 in this design can also be defined as CT1 and 5. In DD samples were collected from CT1 at a 4 hour resolution (n = 3 per timepoint, 8 time-points) (Figure 2A). For the statistical analysis ZT5 to ZT5 was used for diel conditions, and CT9 to CT29 for constant conditions. Collections during the dark phase were conducted under dim red light. During sampling fish were netted out and euthanized by an overdose of benzocaine (150ppm). Weight and length were recorded and no significant variation noted. Optic tectum, gill and saccus vasculous were dissected and snap frozen on dry ice. RNA was extracted for subsequent nanostring profiling.

Circadian experiment II

Fish were maintained in 500L freshwater were transferred from LL to a short photoperiod (SP; 6L:18D) light schedule for 20 weeks before the start of the experiment. Two weeks before sampling, fish were distributed to two separate 150L tanks. Fish were fasted for 48 hours prior to the experiment and throughout the sampling. Temperature was maintained at an average of 8.5oC ±1. Sampling was at a 4 hour resolution starting one hour after lights on (ZT1), maintaining the fish under diel conditions (SP; 6L:18D) for 24 hours and then switching to either LL or DD, and sampling at 4h resolution for a further 29 hours (n = 4 per time-point) (Figure 4A). Collections during the dark phase were conducted under dim red light. During sampling fish were netted out and euthanized by an overdose of benzocaine (150ppm). Weight and length were recorded and no significant variation noted. Blood was collected at each time-point from the caudal vein in heparinized vacutainers. Blood samples were centrifuged at 500 x g for 15 min to collect plasma for subsequent hormone analysis..”.

Page 22 line 432 – 4333: “Following euthanasia whole gill arches were rapidly dissected (biological replicates, n=4 per treatment)…”

Page 35 line 711-718: “A. Diel and circadian regulation experiment design, arrows indicate tissue collection points. Fish were maintained in a light dark cycle (“Diel” - short photoperiod 6 hours light: 18 dark) transitioning onto either constant light (LL) or constant dark (DD). Sampling in diel conditions was at a 4h resolution starting from 5 hours after lights on (Zeitgeber time - ZT5) for 24 hours. The green line represents the time-points used in the statistical analyses of diel rhythmicity. Constant conditions are defined by circadian time (CT) which is relative to lights on in the preceding cycle. The assessment of DD or LL rhythmicity was made using samples from CT9 to CT29, indicated by the red and blue lines. N = 3 for all time-points”.

Page 35 line 720-726: “ Fish were maintained in a light dark cycle (“Diel” - short photoperiod 6 hours light: 18 dark) transitioning onto either constant light (LL) or constant dark (DD). Sampling in diel conditions was at a 4h resolution starting from 5 hours after lights on (Zeitgeber time - ZT5) for 24 hours. The green line represents the time-points used in the statistical analyses of diel rhythmicity. Constant conditions are defined by circadian time (CT) which is relative to lights on in the preceding cycle. The assessment of DD or LL rhythmicity was made using samples from CT9 to CT29, indicated by the red and blue lines. N = 3 for all time-points”.

Page 36 line 734: “ Time-points for sampling (n=6) indicated by arrows.”

Page 36 line 741: “A. Diel and circadian profile of plasma cortisol (n=4)”.

Page 37 line 746-747: “. C. Plasma cortisol concentration in blood plasma in sea-water stress experiment (n=11-12). D. Sea-water stress regulation of clock genes (n=6)”.

Page 37 line 757: “After photoperiod manipulation whole gill arches (n=4) were removed and treated for 24h….”

Page 38 line 7772-3:” A. Osmolality (mOsm kg-1) is displayed for fish in freshwater (FW – blue) and seawater (SW – green) (n=6)”.

We also generally improved the methods description for the smoltification experiment:

Page 222-23 line 423-450:

“Smoltification experiment and seawater tests

Using a standard aquaculture and research method we used photoperiod to induce the developmental transition of juvenile salmon (parr) to seawater prepared fish (smolts). This method requires switching parr on constant light to short photoperiod (SP; 6L:18D) for at least 8 weeks before returning the fish to constant light where over 4 weeks they gain osmo-regulatory capacity in seawater (Figure 3A). Fish were maintained in 150L freshwater tanks at ambient temperature (average 8.5oC) and were transferred from LL to SP (8L:16:D) for 8 weeks before return to LL for 7 weeks. Fish were sampled at six time-points in fresh-water shown in Figure 3A (T1-T6, n = 6 per time-point), these time-points are designed to capture the photoperiodically induced transition from parr to smolt. Fish were fasted for 48 hours prior to sampling. Gills were collected into RNAlater (Sigma-Aldrich, St. Louis, Missouri, USA), storing at 4°C for 24 hours, before being transferred to -80°C. RNA was extracted and used for RNA-seq (Data shown in Figure 3).

To validate the photoperiod protocol seawater challenge tests were conducted on a randomly selected subgroup that were transferred to a 100L tank supplied with seawater (34% salinity) for 24 hours. Fish were netted out after 24 hours and euthanized by an overdose of benzocaine (150ppm). Blood samples were collected from the caudal vein in heparinized vacutainers and plasma was collected and stored at -20℃. To test the osmolality of blood thawed plasma samples were analysed for osmolyte content using a Fiske One-Ten Osmometer (Fiske Associates, Massachusetts, USA, ± 4 mOsm kg-1). These tests were conducted from T2 to T6 (n = 12 per time-point), and confirm that from T5 (29 days after the return to LL) the fish had osmo-regulatory capacity in seawater (S3 Figure). Gills were collected at each time-point after seawater challenge (n= 6 per time-point), RNA was extracted and RNA-seq preformed as above. All data are available from the European nucleotide archive under project number: PRJEB34224. This study presents data from the freshwater groups at all time-points (Figure 3) and seawater challenged fish at T4 only (Figure 4B). T4 is 8 days after return to LL and therefore these fish do not have osmo-regualtory capacity in seawater and are used as a stress test comparison to fish from the same time-point kept in freshwater (Figure 4B – F)”.

*General comments to materials and methods/Figures/results:*

*-why did the authors choose a short photoperiod for all experiments? Why is the constant temperature variable from experiment to experiment? Will this impact gene expression patterns?*

The use of short photoperiods was sufficient for the aims of our circadian study by providing a strong, consistent zeitgeber. It also represents a relevant photoperiod state with implications for industry: under standard aquaculture and research practice, Atlantic salmon are only exposed to either constant light (no circadian information) or SP (to stimulate smoltification).

Our choice of temperature stabilisation reflects the aims of our diverse experiments. Within a permissible range, photoperiod-driven smoltification is independent of temperature. This means that the underlying mechanisms of smoltification persists within this range, and as a result standard smolt production protocols do not typically stabilise water temperature. We adopted these conditions for our photoperiodic experiments. We were, however, concerned that circadian gene expression may be synchronised by temperature directly. We therefore maintained the fish from circadian experiment I at constant temperatures with only a small variation 0.5C observed. Circadian experiment II conducted at a higher temperature but temperature was stabilised with a variation of 1C observed. We observed no effect of temperature in our results.

We have updated the methods section to include this information, see Page 19 – 20 line 368 – 400 (also pasted above).

*-I am not sure I understand how the experiment from Figure 3 was conducted. Did you sample across the day in juveniles or did you sample at the same time of day across multiple days? Did you sample on an LD cycle or in LL?*

We apologise for the confusion and have updated the manuscript to clarify the experimental design, see

Page 222-23 line 423-450:

(also pasted above).

In short, this was an experiment conducted over 110 days to produce a seawater tolerant salmon, it is standard practice to do this by transferring juvenile salmon (parr) from constant light to short photoperiods for 8 weeks followed by return to constant light, whereby within 4-6 weeks they have developed into a seawater tolerant phenotype (smolts) (s3 figure). The sampling was conducted at 6 timepoints over the experiment and at each timepoint the fish were sampled at the mid-light phase (mid-light in LL was based on the time used when the fish were in short photoperiod). So we sampled at the same time of day across multiple days in both and LD cycle and in LL, but the relevant comparison is over seasonal or developmental time, not daily time.

We made additions to the results to clarify:

Page 10 line 1781-183: “We therefore performed a photoperiod manipulation experiment, over 110 days, to assess the impact of photoperiod-dependent developmental changes in juvenile salmon (parr) (Figure 3A, see smoltification experiment, Materials and Methods)."

Page 10 line 179-180:” This developmental transition during the anadromous lifecycle relies on hormonally-driven changes in physiology, dependent on seasonally changing day-length (photoperiod) [34]”

Page 10 line 186-187: “We identified 30 clock genes showing significant changes in expression over the 110 days of the experiment (FDR<0.01, S1 Table, Figure 3B)”

Page 11 line 189-191: “Amongst the differentially regulated genes we found 9 Ss4R pairs (18 genes) where both copies within a pair showed smoltification-associated regulation, while for 3 pairs only one copy of the pair showed significant changes in expression during smoltification”.

Page 11 line 200-201: “Therefore we see strong developmental regulation of clock genes in the gill over a chronic time-scale, contrasting with the lack of circadian regulation”.

Page 11 line 208: “Nevertheless, cortisol is a major hormonal regulator of smoltification in Atlantic salmon, steadily rising during this photoperiod-driven seasonal process [36].”

Page 11-12 line 212-215: “We hypothesized that the changes in gene expression observed in Figure 3 may be due to seasonally increasing cortisol during smoltification resulting in clock gene expression, therefore by inducing cortisol through a simple stress test we could induce the same clock genes seen during smoltification.”.

Page 12 line 220-2122: “Importantly, 87% of acutely SW-responsive clock genes (13/15) also change over the chronic developmental time-scales of smoltification (Figure 3, S1 Table)”.

Page 36 line 733-735: “A. Photoperiodic gene expression experiment design. LL – constant light, SP – short photoperiod. Time-points for sampling (n=6) indicated by arrow, sampling was conducted once per time-point in mid-light phase, time scale shown is experimental days”.

*- line 197 no/weak oscillation in peripheral tissues as seen in 2B – it looks to me like the heatmap has some oscillating clock genes. Or am I interpreting the figure wrong?*

Apologies we have referred to the wrong figure to clearly make our point, Figure 2 b shows a 6 clock genes in the gill and 9 in the sv that have a daily rhythmicity in a light dark cycle, but we only show circadian oscillations (i.e. oscillations that persist in constant conditions, maintaining phase ) in one gene in the sv, see page 9 line 156-158 and supplementary figures 2b-c. We have corrected the manuscript:

Page 11-12, line 211-212: “along with weak or absent peripheral tissue clock gene circadian oscillation (S2B-C Figure)”.

*- Did you collect blood samples pre or post smoltification, or during?*

We collected blood samples throughout the smoltification experiment represented in figure 3, the osmolality data (indicating seawater preparedness and therefore smolting phenotype) can be found in supplementary figure 3. This shows that timepoints T2, T3 are pre-smolting, T4 is a potentially a transition point, and T5 and T6 are post smolting. We have improved the description of this experiment in the methods, see Page 222-23 line 423-450 (also pasted above).

*- In vitro gill culture: why have the authors not included a no-treatment control? DMSO can be toxic and can cause stress responses in cultures, depending on dose. A no-treatment control should be included to exclude effects from DMSO vs drug, or alternatively reference experiments where this has been done.*

The DEX was dissolved in 0.1% DMSO therefore a vehicle control (DMSO) was used, this is standard practice in chronobiology labs and the fairest control, otherwise we would not be able to differentiate the effects of DMSO from DEX. Two examples of the use of DEX and DMSO as a control can be found in Izumo et al (2006) Quantitative Analyses of Circadian Gene Expression in Mammalian Cell Cultures. PLoS Comput Biol 2(10): e136. <https://doi.org/10.1371/journal.pcbi.0020136> and So et al 2009 Glucocorticoid regulation of the circadian clock modulates glucose homeostasis, PNAS, [/doi.org/10.1073/pnas.0909733106](https://doi.org/10.1073/pnas.0909733106) , but there are many more. As far as we know a non-DMSO control is not usually included as standard. We also note that the amount of DMSO we used was 0.1% which is noted to non-toxic to cells, toxicity does not appear until 1% DMSO.

*- The authors talk a lot about mammals vs salmon. I find the analysis a bit shallow and completely void of any general teleost clock knowledge. If you were to look into other fish species like zebrafish or medaka which has undergone one WGD less than the S.salar, you would find that actually, genes in clock loop1 are only slightly greater in number in S.salar compared to D.rerio. This may mean that functionality of the loop1 genes remains the same. The majority of the duplicated genes can actually be found in loop2, which in itself is an interesting observation, and I believe it should be discussed and analysed further. The idea that the different circadian loops might have more molecular and evolutionary “flexibility” is an interesting idea.*

We agree with the reviewer and have updated our manuscript extensively to include better comparison to zebrafish and other teleosts. We did also look at loop 1 vs loop2 gene retention and found no differences We have updated the manuscript with zebrafish relevant information and discussed the idea of “flexibility in the revised manuscript see response to reviewer 1 above and Page 5 line 88-90, Page 7 line 104-106, Page 7 line 110-113, Page 15-16 line 277-301

Furthermore, we used our expression divergence index to compare loop 1 and loop2 genes within the salmon and saw no difference, though perhaps a trend, but not one we feel is report worthy, see below.

*General comment to discussion. Differential regulation of clock genes in different tissues have been described previously in zebrafish though multiple microarray and RNAseq experiments and therefore this find cannot be described as novel. However, it is probably the first of its kind at this sampling frequency and in Salmo salar.*

While we disagree with the reviewers comments that our data is not novel we do concede that we have not represented the zebrafish literature as well as we could. We have therefore updated the manuscript extensively at Page 5 line 88-90, Page 7 line 104-106, Page 7 line 110-113, Page 15-16 line 277-301 (see response to reviewer 1). Additional comments to make are that microarray studies can’t generally resolve between paralogues, so whilst there will have been good circadian studies conducted on zebrafish in a select group of clock genes we do not know of a study encompassing all the known duplicates of clock genes in zebrafish. As the reviewer points out this will certainly be the first of its kind in salmon in sampling frequency but it also in coverage and comparison of the whole duplicated circadian network in a fish.

*Line 265 So far, most fish examined are directly light sensitive and can set their local clocks. The role of an anatomical SCN is not clear, though there is a considerable literature on the role of the pineal gland in regulating locomotor activity and sleep. For example, blocking input from the pineal has an effect on some behavioural traits but not on local tissue clocks. These previous finds would mean that your speculations ‘that this shifting relationship between glucocorticoids and clock genes could be a contributory factor for the evolution of anadromy and the regulation of its seasonal timing’ would not hold up as the peripheral vs central clock organisation in teleostei are different and arose probably long before Ss4R, and earlier down the evolutionary tree of the teleostei.*

From a mouse and zebrafish perspective, the pineal gland would be of interest as it still shows a circadian rhythm in melatonin production in constant darkness, and therefore represents a strongly circadian tissue. However, Iigo 2007 noted that there was no circadian regulation of melatonin release from the pineal gland of seven different salmonids. Also our own unpublished data confirms that in vivo in constant light and dark there is no circadian rhythm of melatonin production in the Atlantic salmon, this is has also been observed in vivo in Arctic charr (a salomind) (Strand 2008). Circadian research in other teleosts (overwhelmingly zebrafish, and where not zebrafish often confusing circadian and diel) has shown that every tissue tested has a tissue autonomous clock that can be directly entrained by light, and that the clock is “paused” in a cell autonomous manner by extended light exposure. In contrast, we show that in the Atlantic salmon (1) the SV and gill have no functional clock and (2) that the strong functional clock in the OT persists in constant light. These data reject a generalised model for the circadian system in fish and highlight the potential diversity of clock mechanisms that likely exist within the teleost clade. We therefore think that the reviewer’s assertion that “the peripheral vs central clock organisation in teleostei are different and arose probably long before Ss4R, and earlier down the evolutionary tree” is unjustified and probably incorrect.

Iigo M, Abe T, Kambayashi S, Oikawa K, Masuda T, Mizusawa K, et al. Lack of circadian regulation of in vitro melatonin release from the pineal organ of salmonid teleosts. Gen Comp Endocrinol 2007 Oct;154(1–3):91–7.

Strand JET, Aarseth JJ, Hanebrekke TL, Jørgensen EH. Keeping track of time under ice and snow in a sub-arctic lake: plasma melatonin rhythms in Arctic charr overwintering under natural conditions. J Pineal Res. 2008 Apr 1;44(3):227–33.

*Line 267-268 ‘Much of the genetic complexity seen in the salmonid circadian clockwork following the Ss4R event; we therefore asked how expression patterns diverged within Ss4R pairs.’ Again, how can you say this when you haven’t compared its complexity to other teleostei that haven’t undergone the Ss4R?*

Our comparison of Ss4R duplicate pairs within circadian, photoperiodic, stress and GR signalling paradigms has conclusively shown that Ss4R was responsible for stimulating great genetic complexity through diversification of pairs within the circadian clockwork. However, we accept that this is a conclusion rather than a way to frame our discussion and so have changed the text to reflect this.

Page 17 line 321-324: “While evidence of functional divergence in ancient duplicated cryptochromes, and, sequence divergence of Ts3R cryptochromes in zebrafish have been observed [60] we expected that Ss4R generated significant genetic complexity in the salmonid circadian clock and therefore asked how expression patterns diverged within Ss4R pairs”.

**Minor comments:**

Lines 52-55 and 55-56. These two sentences contain a lot of information and should be referenced.

We have added references accordingly.

Line 108: comma should be added after ‘duplicated regions’

We have changed the text to better clarify our point:

Page 7 line 114-117: “Following an autopolyploidization, such as the Ss4R, the tetraploid genome will accumulates mutations which blocks recombination and thereby accelerates duplicate divergence (referred to as rediploidization) [24]. Importantly, this process has occurred at different rates in different genomic regions in salmonids”.

*Line 182 ss3r- I think the authors mean ss4r*

Done

*Line 252 Comma after “Nonetheless”*

Done

*Lines 236-237 Why are you not considering other circadian outputs such as cell cycle or immune systems?*

We did not mean to focus just on metabolism in this sentence. We have updated the sentence to include “cellular processes” in order to acknowledge the other outputs without making the sentence too cumbersome:

Page 14 line 253-255: “Ultimately, these differences likely reflect the uneven influence of diel factors on metabolism and cellular processes in central and peripheral tissues in a cold blooded fish”.

*Line 237-238 ‘Altered sensitivity to cortisol emerges as one proximate cause of differences in tissue-specific expression dynamics. The researchers haven’t looked at cortisol sensitivity in different tissues, if this is previous research it should be referenced.*

Ss4R duplicate pairs have altered sensitivity to glucocorticoid receptor signalling, which we correlate with the cortisol axis. We have altered the text to reflect this:

Page 14 line 255-256: “Altered sensitivity to GR-signalling, likely mediated through the cortisol axis, emerges as one proximate cause of differences in tissue-specific expression dynamics”.’

***Reviewer #3***

*The authors have presented a detailed analysis and description of the clock gene network
paralogs present in Atlantic salmon. It describes the tissue distribution and expression
differences between 4R paralogs. I think that the analysis is thorough and well executed with
followup experiments.*

*The main findings of the study is that the salmonid lineage specific whole genome duplication has
provided additional members of clock gene families that have had the possibility to diversify
in expression between different tissues and also in specialise to possibly perform other
functions then regulation of the circadian clock.*

We thank the reviewer for their positive comments and are pleased they see the value of our work.


GENERAL COMMENT

*Is it common practice to use the suffix -ChXX for Atlantic salmon gene names? I suppose it is
referring to the chromosome the gene is located on. If it is not common practise add an
description on this in the manuscript as to not confuse the reader. Alternatively use the
gene name format common to what is used in atlantic salmon.*

We agree that it is not common practice to use chromosome identifiers for Atlantic salmon gene names. We considered following more traditional gene nomenclature however we found that in dealing with duplicates this system quickly becomes difficult to follow – i.e. Nr1d2ba vs Nr1d2bb is more difficult to quickly scan and interpret compared to Nr1d2-Ch14 vs Nr1d2-Ch27. Therefore, we would like to maintain our current gene naming system. In order to clarify to the reader we have added the following explanation and updated S1 table:

Page 7 line 110-113: “ The chromosomal locations of the Ss4R pairs are shown on Figure 1C, and for ease of comparison the genes will be referred to by their gene name and chromosome. The S1 Table lists the specific gene identifiers and orthologues to zebrafish, medaka, spotted gar and mouse”.

*INTRODUCTION*
*on line 59, 77: it is today well established that these two rounds of WGD are common among
at least all jawed vertebrates and not only tetrapods as stated here.*

We have updated Figure 1A to reflect this point more clearly.

Our statement at line 59 is technically correct, we state 2 rounds preceded the establishment of the tetrapod linage 500 million years ago, not that it is only common to tetrapods, we believe along with our improved Figure 1A this is clear. However, we see that line 77 is misleading and have updated accordingly:

Page 5 line 77-79: “Following the two basal vertebrate WGD events (Figure 1A), subsequent rounds of WGD have occurred in several linages, resulting in highly complex genomes containing thousands of ohnologue pairs.”.

*on line 80: It is important to note the 3R WGD is shared by all teleost fish.*

We thank the reviewer for pointing this omission out and have updated the manuscript accordingly:

Page 5 line 79-82: “This is exemplified by the situation found in the salmonids, which underwent two additional rounds of WGD compared to basal vertebrates; a third round WGD (Ts3R) shared by all teleost fish, and a more recent, the salmonid-specific fourth round of duplication (Ss4R) taking place some 100 MYA (Figure 1A) [19].”

*TISSUE EXPRESSION OF 4R PARALOGS*

*Did you consider looking at the expression of these genes in the pineal organ, if so why was it
excluded?*

From a mouse and zebrafish perspective, the pineal gland would be of interest as it still shows a circadian rhythm in melatonin production in constant darkness, and therefore represents a strongly circadian tissue. However, Iigo 2007 noted that there was no circadian regulation of melatonin release from the pineal gland of seven different salmonids. Also our own unpublished data confirms that in vivo in constant light and dark there is no circadian rhythm of melatonin production in the Atlantic salmon, this is has also been observed in vivo in Arctic charr (a salomind) (Strand 2008), therefore we chose not to include this tissue in our study.

Iigo M, Abe T, Kambayashi S, Oikawa K, Masuda T, Mizusawa K, et al. Lack of circadian regulation of in vitro melatonin release from the pineal organ of salmonid teleosts. Gen Comp Endocrinol 2007 Oct;154(1–3):91–7.

Strand JET, Aarseth JJ, Hanebrekke TL, Jørgensen EH. Keeping track of time under ice and snow in a sub-arctic lake: plasma melatonin rhythms in Arctic charr overwintering under natural conditions. J Pineal Res. 2008 Apr 1;44(3):227–33.

*I couldn't find any information in the salmon genome paper from which you have used data
regardning tissue specific expression does not state time of day of collection or how many
individuals were used. Do you have any information about this, that could be added? Could
this affect the results presented you think?*

In the Lien study which performed a multi-tissue expression analysis they used one “adult” male for all tissues. This fish was in fresh water prior to tissue collection. As this was an n of one there is no time of day issue between samples but of course the relative expression of each clock gene in each tissue may be time of day dependent. This study is used as an initial snapshot preceding our main findings which use multiple fish at multiple times of day. As we did not conduct this study we have no further information but we have updated the methods to reflect the sample information:

Page 18, line 353-355: “ Publically available data was used to assess the multi-tissue expression in the Atlantic salmon (one adult male, kept in freshwater), these data can be found in the NCBI Sequence Read Archive (SRA): PRJNA72713 and PRJNA260929”.”

CIRCADIAN EXPERIMENTS

*In the different circadian experiments the short photoperiod light schedule were kept for
different number of weeks (8 and 20), why was it done like this?*

The main purpose of these experiments was to have fish entrained to a light dark cycle – both these designs achieve this. The reason for the difference was on a 3Rs basis; our standard husbandry practices mean that ordinarily fish are maintained on short photoperiod for only 8 weeks before transfer to constant light, this was the basis on which the 1^st^ circadian experiment was conducted. However, as part of a related study fish were maintained on short photoperiods for longer, in these experiments a large number of “dummy” fish were required to fulfil welfare requirements. Therefore, we chose at the end of that related study to make use of the “dummy” fish. This meant that 77 fish, which would have not be used in the related study, were in fact used for a scientific purpose. 20 weeks of short photoperiod does mean that there is a difference in the age of the fish between the first and second experiments but as the goal was entrainment to a light dark cycle and we are not directly comparing the two experiments we do not believe this is an issue. Also 20 weeks of short photoperiod is unlikely to cause any issues as in the wild these fish will experience at least that duration of short photoperiod (i.e. below 12 hours). To clarify the experimental design we have updated the methods section accordingly. See response to reviewer 2 and these specific lines of the manuscript: Page 19 – 20 line 368 – 400

*In the method section i cant find the intervals and timepoints for sampling and why they
were chosen, i would recommend adding this to the materials and method section.
Im not sure i understand the figures showing this since not all times are added and its
not clear when the start time is. Figure 4A is much clearer in this regard.*

We have updated the methods extensively, see response to reviewer 2 and these lines of the manuscript: Page 19 – 20 line 368 – 400, Page 22 line 432 – 433, Page 35 line 711-718, Page 35 line 720-726, Page 36 line 734, Page 36 line 741, Page 37 line 746-747, Page 37 line 757, Page 38 line 772-3, Page 222-23 line 423-450

In addition we have updated Figure 2A to represent the times more clearly. We first show the zeitgeber time (time since lights on (ZT)) and the ZTs for sampling in the light dark cycle (Diel). We then show the circadian time (time since constant condition began (CT)) and the CTs for sampling in either DD or LL. We have also clarified the figure legend:

Page 35-36 line 720-726: “A. Diel and circadian regulation experiment design, arrows indicate tissue collection points. Fish were maintained in a light dark cycle (“Diel” - short photoperiod 6 hours light: 18 dark) transitioning onto either constant light (LL) or constant dark (DD). Sampling in diel conditions was at a 4h resolution starting from 5 hours after lights on (Zeitgeber time - ZT5) for 24 hours. The green line represents the time-points used in the statistical analyses of diel rhythmicity. Constant conditions are defined by circadian time (CT) which is relative to lights on in the preceding cycle. The assessment of DD or LL rhythmicity was made using samples from CT9 to CT29, indicated by the red and blue lines. N = 3 for all time-points”.

Figure 4a is easy to display in this regard because sampling began at ZT1 and there is no extra information regarding the statistical analysis to convey in figure 4a.We hope our new figure and text updates clarify matters.

*In the materials and methods section I cant find information about the number of individuals
were sampled per experimental condition/time-point.*

We apologise for the omission and have extensively up dated the manuscript methods and legends to reflect this information.

We have updated the methods extensively, see response to reviewer 2 and these lines of the manuscript: Page 19 – 20 line 368 – 400, Page 22 line 432 – 433, Page 35 line 711-718, Page 35 line 720-726, Page 36 line 734, Page 36 line 741, Page 37 line 746-747, Page 37 line 757, Page 38 line 772-3, Page 222-23 line 423-450

*DISCUSSION
How does the data presented here for the atlantic salmon fit in the context of expression
data presented in the literature from other species of salmonids?
There seem to be publications regarding the expression of some of the clock genes in
north american salmonids.*

Whilst we accept that there are some studies on a limited number of clock genes in other salmonids often these studies have not completely accounted for zeitgebers and/or have presented data only from diel conditions. In our manuscript we provide S3 table which illustrates the variability in the design of experiments on circadian function in salmonids (and other fish) which make definitive statements regarding the persistence of circadian endogenous rhythms in salmonids difficult to make. Furthermore these studies have not resolved between ss4R duplicates and directly compared the expression of duplicates. There is no comparable data from the SV or gill but there is diurnal expression data of Arntl-Ch10, Clock-Ch23, Cry1-Ch22, Per1-Ch7 and Per2-Ch14 in the salmon brain (Davie et al 2009, Huang et al 2010a/b). Our data is consistent with the published diurnal oscillations but there are differences in peak phase, this is likely due to the presence of other zeitgebers such as food and temperature in the previous studies which we controlled for. Whilst Huang et al (b) also measured gene expression under LL noting no oscillations, these fish were kept in LL for six weeks before sampling which means any persistent circadian rhythms that may have been present would have most certainly been dampened by then. Therefore, interpretation of previous work is difficult due to the lack of control over the zeitgebers of food and temperature. The zebrafish literature is much more extensive and accounts for zeitgebers therefore comparison to this literature appears to be more relevant. We have therefore updated our manuscript accordingly:

Please see response to reviewer 1 above and Page 5 line 88-90, Page 7 line 104-106, Page 7 line 110-113, Page 15-16 line 277-301 in the manuscript.

Davie A, Minghetti M, Migaud H. Seasonal Variations in Clock‐Gene Expression in Atlantic Salmon ( Salmo salar ). Chronobiol Int. 2009;26: 379–395. doi:10.1080/07420520902820947

Huang T, Ruoff P, Fjelldal PG. Diurnal expression of clock genes in pineal gland and brain and plasma levels of melatonin and cortisol in Atalantic salmon parr and smolts. Chronobiol Int. 2010;27: 1697–1714. doi:10.3109/07420528.2010.514630

Huang T, Ruoff P, Fjelldal PG. Effect of continuous light on daily levels of plasma melatonin and cortisol and expression of clock genes in the pineal gland, brain, and liver in Atalantic salmon postsmolts. Chronobiol Int. 2010;27: 1715–1734. doi:10.3109/07420528.2010.521272

*Did you observe any functional specialisation between paralogs resulting from 3R in those
cases where you didn't find any differences between the 4R paralogs?*

There is evidence for differences at the Ts3R level when differences are not clear at the Ss4R level. For example, Hlf-Ch3/6, and Hlf-Ch1/28 (both Ss4R pairs) are co-regulated but as viewed from the Ts3R event the pairs have clear differences – Hlf-Ch3/6 are photoperiodically regulated; Hlf-Ch1/28 are not. In this study we were specifically interested in ss4R pairs because of the association with anadromy and their relatively recent appearance in evolutionary time makes estimates of divergence time and evolutionary relationships easier to resolve and more accurate compared to older duplication events. Furthermore, we expected that ss4R pairs would be the greatest contributors to the genetic complexity of the salmonid circadian clockwork. So while we present data from all clock genes (S1 table etc) our manuscript mainly focuses on the ss4R pairs.

*Has functional divergence of 3R duplicates been described for other teleost species?*

In relation to clock genes the best evidence for functional divergence of 3R duplicates is found in the cryptochromes of zebrafish (lui 2015). These authors show that after Ts3r zebrafish retained 6 cry genes which show different expression patterns in a light dark cycle and some evidence of tissue specific expression by whole mount in situ. However comparisons in a circadian series were not undertaken and all show rhythmicity under a light dark cycle. These authors also provide in vitro evidence that the crys can be divided into repressive or non-repressive crys – suggesting functional divergence. It is interesting to note that the authors report that the different functions may be attributal to a more ancient local duplication event rather than WGD. However, they do observe sequence divergence in the Ts3R duplicate cry1ba/cry1bb.

Liu, C., Hu, J., Qu, C. et al. Molecular evolution and functional divergence of zebrafish (Danio rerio) cryptochrome genes. Sci Rep 5, 8113 (2015). <https://doi.org/10.1038/srep08113>

We have updated the manuscript:

Page 17 line 321-324: “While evidence of functional divergence in ancient duplicated cryptochromes, and, sequence divergence of Ts3R cryptochromes in zebrafish have been observed [60] we expected that Ss4R generated significant genetic complexity in the salmonid circadian clock and therefore asked how expression patterns diverged within Ss4R pairs”.

FIGURES
*Figure 1A: Why is there no root in this tree, and what does the strange branching between
coelacanth and terapods mean?*

Figure 1A has been updated to be clearer and more representative.

*Figure 1C: What does the lines that are not for the clock genes represent in this figure?
Is it possible to have different colors for the lines representing different clock 4R paralogs?*

The lines that are not for clock genes represent homologous (duplicated) regions of the salmon genome, therefore large collinear blocks of the genome are duplicated on different chromosomes, in which ss4r duplicated clock genes are found. We have updated the figure to make all the collinear blocks grey and have revised the line colour between ss4r ohnologue clock genes to represent the orthogroups. We felt that a colour for every geen pair would be too confusing and hope that this revision makes the pair relationships clearer. We have also updated the legend for clarification:

Page 35 line 705-710: “C. Circos plot representing the Atalantic salmon chromosomes and the large collinear blocks of the genome that are duplicated (grey blocks/lines). Overlayed are the chromosomal positions of all Ss4R clock ohnologues, duplicated clock genes are connected by a coloured line. The colour of this line represents the different orthogroups. Around the outside of the circos plot sequence similarity of the loci in a 1mb window is shown as a coloured bar; high similarity >95% (red), medium 95-90% (blue), low ~87% (green)”.

*Figure 2:*

*What are the normalised counts, which unit?*

We have updated the methods:

Page 23 line 456-461: “This technology is based on the use of fluorescent barcoded probes which bind specifically to the target molecule. Importantly these barcodes should only bind one at a time to each target molecule, therefore the number of fluorescent barcodes reflects the number of RNA molecules of your target gene. A spike control with a known number of RNA molecules was also used to normalise across samples and runs. Therefore the units are counts normalised to spike-in positive controls”.

*Why is there two scales in figure 2D for Arntl-ch10 Arntl-ch16 in optic tectum. Looking at the
graph it seems better to use the scale to the right for both.*

We felt this more clearly represented the data, however we have updated the graph on figure 2d to only use the right hand axis scaling.

*Figure 3:*

*Looking at the timeline in A, i see that the sampling time intervals differ a lot. Why is
the intervals of equal lengths in B and C. This changes the look of the graphs quite a lot
and could lead to missinterpretation.*

We have updated figure 3 to better represent the time intervals. All axis have been updated. The data appear to be similar and therefore no new interpretation of the results is required.

*SUPPLEMENTARY FIGURES*

*S1 Appendix: For the phylogenetic trees i would suggest adding support values on the nodes.
This would help the reader interpret the results better.*

We included maximum likelihood gene trees with bootstrap values for all circadian pathway genes discussed in this manuscript (S1 appendix). Furthermore we have included more information in the methods section and added a link to the scripts and input data used in this analysis (<https://gitlab.com/sandve-lab/salmon-clockgenes/-/tree/master/>).

Page 18 line 341-347: “Maximum likelihood gene trees with bootstrap values for all genes included in the study are presented in S1 appendix. These trees include the following species: human, mouse, spotted gar, zebrafish, stickleback, medaka, northern pike, grayling, danube salmon, arctic charr, coho salmon, rainbow trout, Atlantic salmon. The relevant scripts, input files and resulting trees are also available [here](https://gitlab.com/sandve-lab/salmon-clockgenes/-/tree/master/). For quick reference S1 table presents the salmon gene loci and their corresponding orthologues in Zebrafish, spotted gar, medaka and mouse”.
