# Supplementary material for: Diversified regulation of circadian clock gene expression following whole genome duplication
Source: PLoS Genet. 2020 Oct 8;16(10):e1009097. doi: 10.1371/journal.pgen.1009097 (PMC7575087; doi:10.1371/journal.pgen.1009097)
Supplement: Attachment — Submitted filename: Reviewers_comments-response-26082020.docx [file pgen.1009097.s015.docx]

**Response to reviewers: PGENETICS-D-20-00531- Diversified regulation of circadian clock gene expression following whole genome duplication**

Reviewer comments are in *italics* and responses are in red.

We thank all reviewers for their comments and input which have already improved the manuscript, we respond to the two outstanding minor comments below.

***Reviewer #1:***

*The revised version of the manuscript has been much improved as a result of addressing this reviewer‘s comments as well as those of the other reviewers. In particular, introducing a comprehensive overview of previous relevant work that has been based on the zebrafish has helped to better place the current study into a more general context. However, it is important that the authors now correct an error that has been introduced with the new text.*

*Page 15-16 line 277-301*

*The authors now make reference to the paper, Pagano et al (Scientific Reports, ref. 55) which they state illustrates how the D-box enhancer in the context of the Tef promoter, mediates transcriptional inducibility by light, UV and ROS. I have just checked the details of this work, and it seems that the D-box that is studied is in the context of the Cry1a promoter, NOT the tef promoter. The authors should correct their text accordingly.*

We are glad the reviewer thinks the manuscript is improved and better placed in the relevant context. We thank the reviewer for pointing out the error and note it was a combined issue of mis-citation and an error in gene name. We have now clarified this on page 15-16 lines 284-296:

“For example zebrafish Cry1a is acutely light-induced through a D-box in it’s promoter, via reactive oxygen species (ROS) and the JNK/MAPK pathway [55]. Importantly this light-mediated ROS inducibility of zebrafish Cry1a appears to be absent in cave fish and mammals [55]. Zebrafish Cry1a has also been reported to pause the circadian mechanism under LL [9], whereas the closest salmon orthologues to zebrafish Cry1a, Cry1-Ch10 and Cry1-Ch16, are not light-induced and show persistent rhythmicity in the OT under LL (S1 Table). Instead the salmon orthologues to zebrafish Cry3b, Cry3-Ch22 and Cry3-Ch12, are acutely induced by light and salt, respectively. Light induction of Zebrafish Tefa [33] is not seen in the closest salmon orthologues Tef-Ch1 and Tef-Ch28, however, the zebrafish Tefb orthologues, Tef-Ch3 and Tef-Ch6 are seawater induced. In zebrafish, Tefb transcription is induced in response to light and UV exposure and then mediates the expression of other clock genes, initiating a DNA damage repair cascade [56]. This suggests a zebrafish-specific adaptation to UV light exposure, potentially related to spawning environment [56]”.

***Reviewer #2:***

*Thank you for updating and improving your manuscript. I believe considerable improvements have been made.*

Thank you for your input in improving the manuscript and we are pleased you feel the manuscript is improved.

***Reviewer #3:***

*I am happy with the answers i received from the authors regarding my comments on the manuscript.*

*I have one remaining minor comments on the revised version. In figure 1A: I think you missunderstood my comment regarding the branching of the coelacanth. It was placed in the correct position in the original figure but the branching was not the traditional bifurcating branching so i wondered why you choose that way to display the sarcopterygian clade (human, mouse), coealanth) while (coho salmon, rainbow trout), arctic charr) were displayed in the traditional way.*

The choice for the original figure was mostly an aesthetic one. After reading your comment we decide to make the branching consistent for the whole figure to avoid any differences that may be assumed to convey additional information when that was not the goal of the figure. Additionally one of the other reviewers commented on the introduction text to clarify when WGDs occurred, therefore we revised figure to better represents when WGDs have occurred and the traditional branching worked best for this.
